# Strong Proper C–H Hydrogen Bonds: Experimental Evidence across Spectral Ranges

**DOI:** 10.1021/jacsau.6c00326

**Published:** 2026-05-05

**Authors:** Anton Kliuchynskyi, Aritri Biswas, Andrey Shalit, Alexei A. Kananenka, Bogdan Dereka

**Affiliations:** 1 Department of Chemistry, 27217 University of Zurich, Zurich CH-8057, Switzerland; 2 Department of Physics and Astronomy, 5972 University of Delaware, Newark, Delaware 19716, United States

**Keywords:** hydrogen bond, electric field, nonelectrostatic effects, terahertz (THz) spectroscopy, infrared (IR) spectroscopy, NMR spectroscopy

## Abstract

C–H bonds are known as weak hydrogen bond (H-bond) donors, and the H-bonds that they typically form are improper: C–H bond shortens, the frequency of its vibrational transition blueshifts, and intensity decreases. All of these characteristics are opposite to familiar proper H-bonds. Here, we demonstrate that an *sp*-hybridized terminal alkyne (≡C–H) is a potent proper hydrogen bond donor. Tuning across >50 solvation environments, we demonstrate the propensity of this moiety to form H-bonds with heteroatoms in polar solvents, aromatic π-systems, and, most surprisingly, even with single π-bonds in nonpolar molecules. We directly observe the H-bond formation and comprehensively characterize it using a combination of infrared (IR) absorption, ^1^H and ^13^C NMR spectroscopies, and broadband time-domain terahertz spectroscopies. Experimental results are interpreted using *ab initio* molecular dynamics simulations. Both electric field effects and nonelectrostatic short-range interactions determine the frequency of the hydrogen-bonded ≡C–H stretch. Based on purely experimental observables, we propose a method to disentangle and quantify these contributions. We also show that a non-hydrogen-bonded ≡C–H is a powerful sensor of dispersion interactions via its IR shift even in the presence of much stronger competing interactions. Overall, the alkynyl ≡C–H mimics an O–H group rather than resembling a typical aliphatic C–H. Its distinct local character and spectral isolation from other C–H stretches, high electric field sensitivity, strong propensity toward H-bond interactions, compactness, and ease of incorporation into molecular scaffolds make this overlooked vibrational marker a strong contender for sensing.

## Graphical abstract

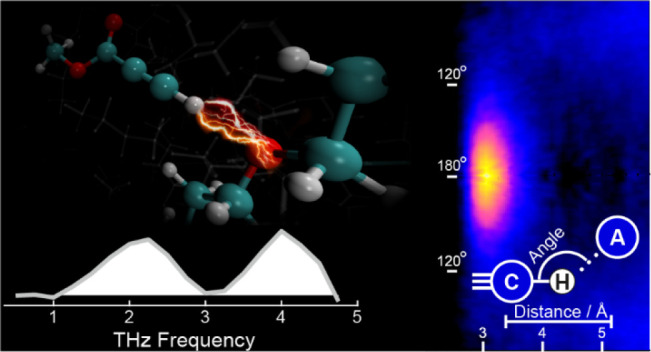

## Introduction

Fine-tuning of noncovalent intermolecular interactions is increasingly recognized as the ultimate tool for precise engineering of properties and behavior of matter. Hydrogen bonds (H-bonds, HBs) represent the most important class of specific interactions underlying properties of water, aqueous solutions, biomolecules, and functional materials. They have been studied for more than a century since Lewis coined the term “hydrogen bond” in his landmark publication in 1923,^1^ where he wrote *“Hydrogen when attached firmly to a pair of electrons, as in the ··· hydrogen–carbon bond, shows no tendency whatsoever to become bivalent, or in other words, to form a hydrogen bond. On the other hand, when combined with an extremely negative element like nitrogen, oxygen or fluorine, toward which the electron pairs are very tightly drawn, ··· the hydrogen atom can form a loose attachment to another pair of electrons, thus forming the hydrogen bond.”* Indeed, the vast majority of research focused on conventional H-bond donors X–H, where hydrogen forms a covalent bond with highly electronegative atom X, while unconventional donors constituting weakly or nonpolar bonds, such as C–H, which, Lewis mentioned explicitly, have not been recognized as hydrogen bond donors for a long time. Now, the role of C–H hydrogen bonds is getting increasing attention in the context of fundamental understanding of the properties of the water–oil interface,^2–5^ in rational design of new materials,^6,7^ (bio)­molecular recognition,^8–10^ supramolecular assembly,^11–13^ and catalysis,^14,15^ among others.

Nevertheless, while C–H moieties are acknowledged for their ability to form HBs, they are not perceived as potent H-bond donors. The reason is that when C–H bonds engage in hydrogen bonding they form very weak HBs that were termed improper hydrogen bonds.^16^ Opposite to proper H-bonds, improper hydrogen bonding leads to shortening of a C–H bond causing a blueshift of the C–H vibrational frequency and loss of its oscillator strength.^17^ There has been a strong interest from the computational chemistry community to unravel the origin of proper/improper HBs by adapting intuitive chemical bonding theory concepts, such as hybridization and hyperconjugation,^17,18^ or dissecting HB energy into physically meaningful terms, such as dispersion, induction (polarization and charge transfer), Pauli repulsion, and permanent electrostatics.^16,19^ In contrast, experimental efforts have been severely lagging behind with very limited reports detailing the nature of improper C–H HBs^11,20–23^ in liquid solution at room temperature and essentially no evidence of strong proper ones.^24^

It is well-known that carbon electronegativity increases upon increasing the *s*-character of the *sp* ^*n*^-hybrid orbitals. As such, *sp*-hybridized is the most electronegative carbon type, and terminal alkynes featuring a single ≡C–H bond are promising candidates for exploring C–H hydrogen bonding.

Recent work by Boxer and co-workers has spotlighted alkynyl C–H bonds as hydrogen bond donors.^24^ In their seminal study, they reported substantial 50–100 cm^–1^ solvatochromic redshifts of the alkynyl C–H stretch of 1-hexyne in 4 oxygen-containing solvents that were ascribed to hydrogen bonding. They elegantly showed that bond polarization and charge transfer play a role in determining the vibrational frequency of the alkynyl C–H stretch.

In this work, we reveal distinctive and remarkable hydrogen bonding properties of alkynyl C–H bonds that have not been reported before. As a prototypical H-bonding probe of this kind, we use methyl propiolate (**MP**) – a methyl ester of propiolic (acetylenecarboxylic) acid (Chart 1, safety information in Section S1.2), which connects a terminal alkyne fragment to the electron-withdrawing carboxylic moiety, which further enhances hydrogen-bond donating properties of the ≡C–H fragment.

**Chart 1:**
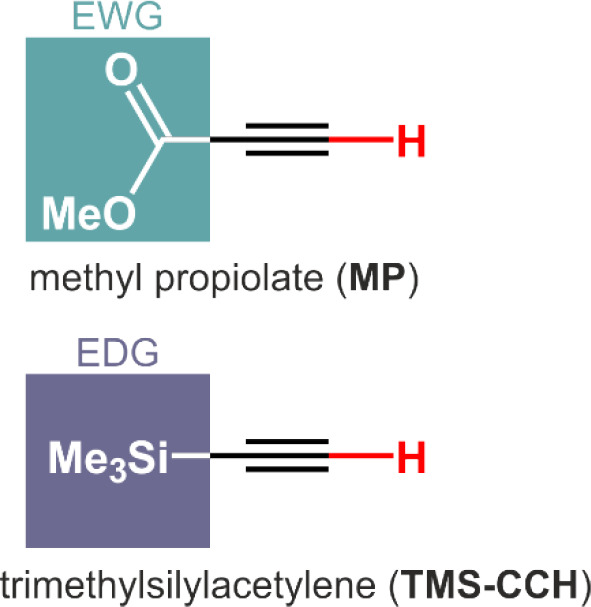
Chemical Structures of **MP** and the Control Compound **TMS–CCH** ^a^

a Electron-withdrawing (EWG) and donating (EDG) groups at the alkyne moiety are highlighted. **TMS–CCH** was used to demonstrate that the ester group in **MP** is not a prerequisite for the hydrogen bonding to occur but boosts its strength.

Using infrared (IR), ^1^H and ^13^C nuclear magnetic resonance (NMR), and broadband subpicosecond time-domain terahertz (THz) spectroscopy, we comprehensively probe ≡C–H···**A** hydrogen bonding across a multitude of spectral ranges and >50 solvation environments, focusing on nonpolar, dipolar, and aromatic solvents. We demonstrate that the ≡C–H fragment is a potent HB donor and is an overlooked vibrational marker whose frequency is strongly sensitive to the electric field and specific interactions with its local solvation environment. We report a striking ability of ≡C–H to form HBs with aromatic π-systems and even single π-bonds in nonactivated apolar molecules, such as cyclohexene or cyclohexadienes. Our experimental findings are supported by *ab initio* molecular dynamics (AIMD) simulations, which characterize the geometric features of HB distributions in liquid solution and reproduce the spectral signatures of hydrogen bonding observed in the THz region. For non-hydrogen-bonded ≡C–H, we show its exquisite ability to sense dispersion interactions, even in the presence of much stronger competitors.

Furthermore, using a combination of IR and NMR spectroscopies and validated by AIMD simulations, we provide an experimental recipe for using ≡C–H to quantify microscopic electric fields and separate electrostatic versus nonelectrostatic effects.

Finally, like other conventional strong H-bond donors (e.g., O–H), C–H in **MP** spontaneously exchanges to deuterium in D_2_O without any catalysts. Altogether, this work demonstrates that proper strong C–H···**A** hydrogen bonds not only exist but, unexpectedly, they behave similarly to their canonical counterparts, such as O–H···**A**. These results have broad implications for our understanding of the structure and reactivity of alkyne-containing molecules.

## Results and Discussion

### Alkynyl C–H Is an Unappreciated Proper Hydrogen Bond Donor

A C­(*sp*)–H stretching vibration of terminal alkynes is a distinct local mode that occurs at ∼3300 cm^–1^.^25^ It is decoupled from all other lower-frequency aliphatic (C­(*sp* ^3^)–H, ∼2900 cm^–1^) and aromatic (C­(*sp* ^2^)–H, ∼3050 cm^–1^) stretches. Indeed, the IR spectrum of methyl propiolate in noninteracting cyclohexane (CHX) contains a sharp band at 3308 cm^–1^ (Figure 1a). This band redshifts with increasing polarity of the environment – a solvatochromic effect that for other vibrations is often interpreted in terms of the vibrational Stark effect (VSE) imposed by the microscopic electric fields of the surrounding environment.^26^ However, we observed that in several ether solvents (Figure S11), the band does not merely shift but splits into two: a sharp band of the free C–H remains near 3310 cm^–1^, and a new intense broad band emerges near 3200–3220 cm^–1^ (see di-*n*-butyl ether in Figure 1a, c). The strong ∼100 cm^–1^ spectral change is a clear indication of an additional and markedly different population of the alkynyl C–H vibrations that coexists with the free C–H band. The shifts of this magnitude were observed in ref 24 in the IR spectrum of 1-hexyne in DMSO and were attributed to the hydrogen-bonded C­(*sp*)–H stretch. Compared with the better understood O–H stretch, this behavior is not straightforward for two main reasons. First, C–H is a notoriously weak HB donor due to the low polarization of this bond – a consequence of nearly equal electronegativities of carbon and hydrogen atoms. Second, even when C–H bonds participate in hydrogen bonding, the common view is that they usually engage in the so-called improper hydrogen bonds that are characterized by weak blue shifts of their respective IR resonances. As such, the strong redshifting nature of the C­(*sp*)–H stretch is highly unusual and deserves further investigation.

**Figure 1:**
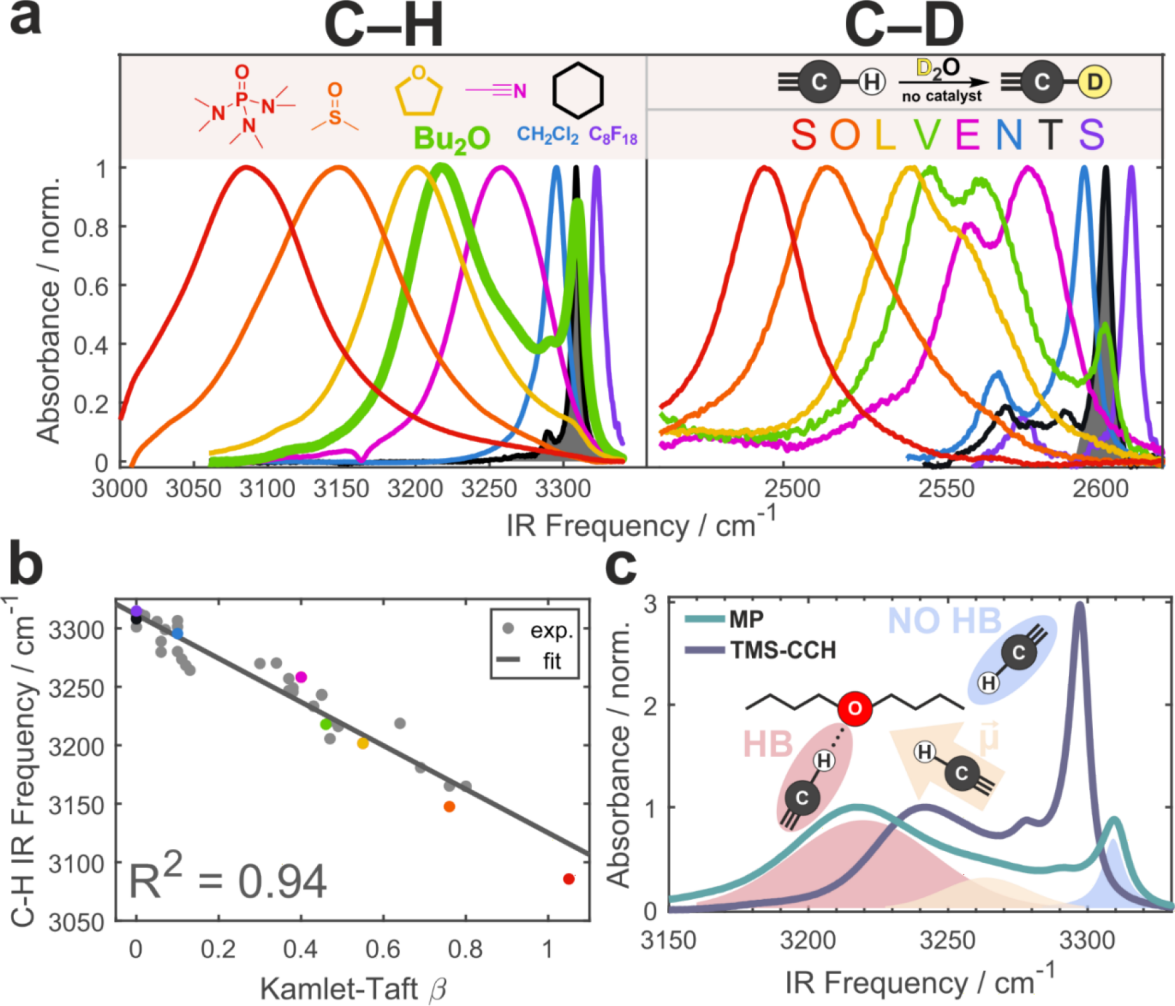
C–H proper H-bonding in terminal alkynes. (a) IR spectra of **MP** ≡C–H (left) and of **MP-d** ≡C–D (right) stretch in selected solvents whose chemical structures are shown. Note the ≡C–H multiband structure in di-*n*-butyl ether (thick green line). **MP-d** is obtained via spontaneous H/D exchange, as shown on top. (b) Dependence of the ≡C–H stretch frequency of **MP** on the H-bond accepting ability of the solvent as quantified by the Kamlet–Taft β parameter. Colored dots correspond to the solvents from (a). Linear fit and its coefficient of determination (*R* ^2^) are shown. (c) IR spectra of **MP** and control **TMS–CCH** in di-*n*-butyl ether. While the H-bond in **MP** is stronger, a qualitatively similar behavior is observed for **TMS–CCH**. Decomposition of the **MP** spectrum into the bands of distinct species of non-H-bonded, H-bonded, and frustrated species is color-coded.

The hydrogen-bonded population becomes a dominant one in highly polar and strongly hydrogen bond accepting media, where the shifts can reach several hundreds of cm^–1^. For example, in dimethyl sulfoxide (DMSO) and hexamethylphosphoramide, the peak of the band shifts to 3148 and 3085 cm^–1^, respectively (Figure 1a). Such a shift of up to 225 cm^–1^ from CHX for the vibration of a nominally apolar C–H bond is remarkable and has not been reported before. Unexpectedly, the C­(*sp*)–H stretch outperforms those of the classical highly polar local modes such as C=O (shifts up to 100 cm^–1^) and C≡N (up to 30–50 cm^–1^) stretches, even when they are subject to strong electric fields or engage in very strong hydrogen bonds.^27–38^ Such a large shift is comparable to the well-studied O–H stretch band.^39–42^ A strong support for the assignment of the observed large shifts to hydrogen bonding comes from a near-quantitative dependence of the C–H band peak on the HB accepting ability of the solvent quantified by an empirical Kamlet–Taft β parameter (Figure 1b).^43^ Quantum-chemical calculations of several H-bonded **MP**-solvent complexes reproduce the experimental trend and the magnitude of the shift (Supporting Information, Section S2.1). Moreover, while the attachment of the alkynyl moiety to the electron-withdrawing ester group polarizes the C­(*sp*)–H and strengthens the H-bond, this behavior is not unique to **MP**. Figure 1c demonstrates that even for apolar (CH_3_)_3_Si–C≡C–H (**TMS–CCH**), the hydrogen-bonded C­(*sp*)–H stretch band shifts by an impressive ∼60 cm^–1^. These examples highlight ≡C–H as a potent and underappreciated proper HB donor.

Another hallmark of strong, proper HBs is their spontaneous H/D exchange upon exposure to deuterated protic solvents. Previous studies reported base-catalyzed ≡C–H to D exchange occurring on many minutes to hours time scales.^44–46^ We found that **MP** exchanges its alkynyl proton to deuterium within seconds upon exposure to D_2_O with no catalysts. We isolated pure deuterated **MP** (**MP-d**) as discussed in Section S1.3. Its IR spectrum demonstrates no C­(*sp*)–H absorption in the 3000–3350 cm^–1^ region, but instead, a new band rises at 2470–2620 cm^–1^ that corresponds to the ≡C–D vibration (Figure 1a). Its frequency is higher than the value based on the reduced mass change due to the coupling of C≡C and C–D stretch vibrations.^44,46^ Similar to protiated **MP**, **MP-d** engages in hydrogen bonding with solvents demonstrating a reduced but still a large >140 cm^–1^ range of frequency shifts depending on its environment.

In contrast, **TMS–CCH** does not exchange its alkynyl proton under the same conditions, indicating a weaker H-bond. Therefore, ≡C–H can be switched between exchangeable or nonexchangeable states by varying the electronic nature of the alkyne substitution. This tunability is highly beneficial compared to the O–H stretch, which is always exchangeable, as it allows facile probing of both protic and nonprotic environments without the need for isotopic dilution or ultrathin samples.

The NMR ^1^H chemical shift (δ^1^H) is a widely used marker as the downfield shift of the proton resonance is a hallmark signature of hydrogen bonding. We performed proton NMR experiments on **MP** in the same solvents as those for the IR measurements. Indeed, the alkyne proton undergoes a notable shift of >3 ppm from 2.55 to 5.79 ppm upon going from perfluorohexane to hexamethylphosphoramide (Figure 4a) in accord with the insight on HB formation from IR spectroscopy (Figure 1a).

### Aromatic π-Systems Accept H-Bonds from ≡C–H

In conventional dipolar solvents, we observed a distinct splitting of the C–H stretch band into sizable HB and non-HB populations in esters, noncyclic ethers, and thioethers – sulfides (Figures S11–S13). However, we noted similar spectral features in aromatic solvents, advocating for the potential involvement of the aromatic π-system as an H-bond acceptor. To investigate this, we systematically varied the hydrogen-bond accepting ability of aromatic systems by modulating the electron density in their π-systems through substitutions in the ring. Substituents that could compete with a π-system as hydrogen bond acceptors were deliberately avoided. As such, we resorted to methyl groups increasing their number from zero (benzene) to one (toluene), two (*p*-xylene), and three (mesitylene). Due to the electron-donating effect of −CH_3_ groups, increasing their number on the core makes it more electron rich and activates the π-system for H-bonding. To deactivate the ring, we used bromobenzene, where a Br atom withdraws the electron density. Being a very weak HB acceptor, it is not expected to outcompete the aromatic π-system.

The strongest effect was observed in mesitylene, where the IR spectrum consists of two overlapping but clearly distinguishable bands – free C–H at 3305 cm^–1^ and the H-bonded C–H at 3264 cm^–1^ (Figure 2a). Band splitting becomes progressively smaller and more difficult to resolve in xylene, toluene, and benzene. In these solvents, increased spectral intensity at 3305–3320 cm^–1^ can be traced via band shape analysis to the free C–H in addition to the main peak of the hydrogen-bonded C–H at lower frequencies. In bromobenzene, the sole band is red-shifted and no band-splitting or shoulder formation can be inferred. Bromobenzene represents a boundary case where no unambiguously observable C–H hydrogen bond formation to the π-system takes place. Due to the dipolar nature of this solvent, shift of the non-H-bonded C–H to 3289 cm^–1^ is attributed to the electric field effect of the environment due to the VSE. As a control, we report no HB formation to the strongly depleted π-system in perfluorinated benzene, while its nondipolar nature shifts the spectrum back to where it is observed in cyclohexane (Figure 2a).

**Figure 2:**
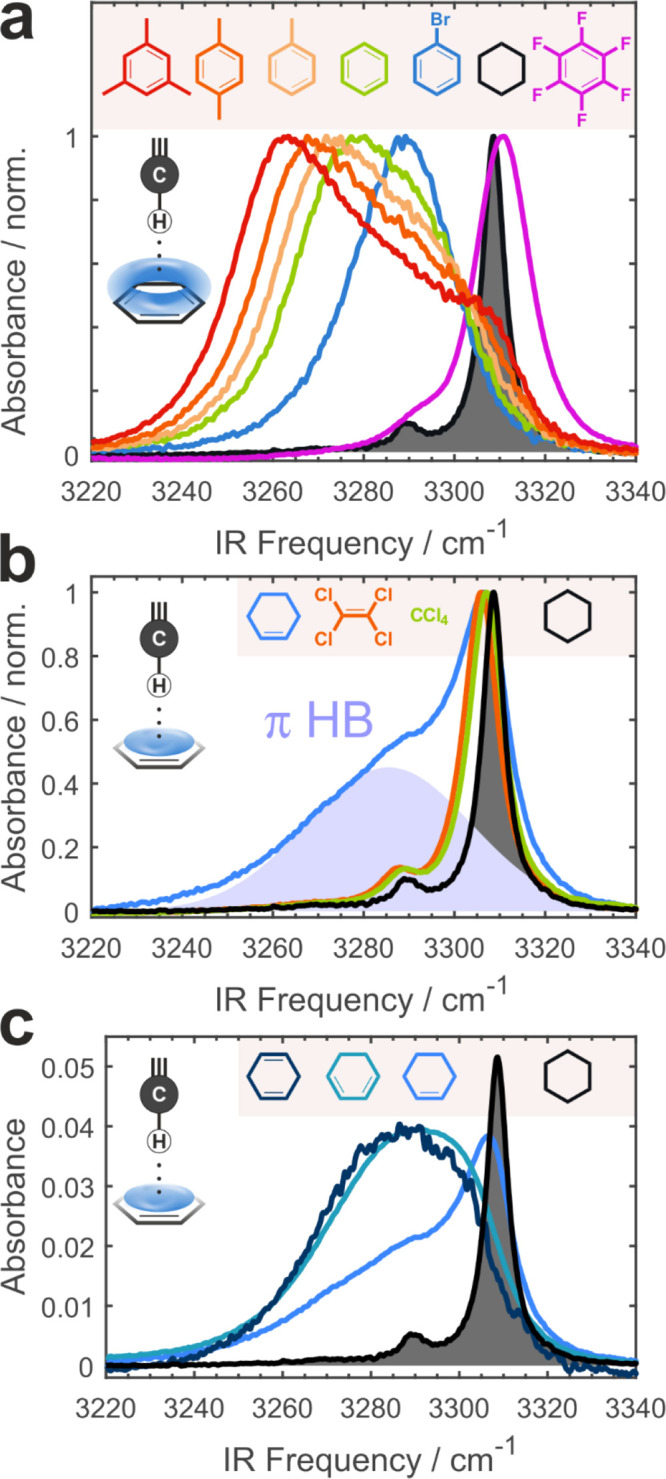
C–H···π H-bonding. (a) IR spectra of the **MP** ≡C–H stretch in aromatic solvents. (b) C–H-single π H-bonding of **MP** to cyclohexene. Deconvoluted π H-bond band is shaded in blue, and reference spectra in perchlorinated methane and ethylene are shown as their free C–H peaks at the same position as in CHXene make the Fermi resonance condition identical. Therefore, the Fermi resonance perturbation cannot explain the new π band. (c) C–H···π H-bonding to a single (CHXene) vs two (CHX2ene) double bonds in conjugated and decoupled cyclohexadienes.

Unlike IR, NMR provides unique spectroscopic evidence of ≡C–H···π-aromatic hydrogen bonding. The proton H-bonded to the aromatic π-system experiences shielding observed as the upfield shift of its resonance. The δ^1^H of ≡C–H shifts significantly (>0.5 ppm) upfield in the aromatic solvents in comparison to noninteracting saturated hydrocarbons, or more when comparing to dipolar H-bond accepting solvents.

### Elusive and Unconventional ≡C–H Hydrogen Bonding to a Single π-Bond

Cyclohexene (CHXene) represents the most extraordinary case and warrants a separate discussion. Cyclohexene differs from cyclohexane by a single carbon–carbon π-bond. Yet, Figure 2b shows how markedly different are the **MP** C–H IR spectra in cyclohexene versus cyclohexane: while the free C–H band is clearly visible around 3305–3310 cm^–1^ in both, a new broad band appears at <3300 cm^–1^ in CHXene. We henceforth refer to this band as “π band”. This splitting into two distinct bands indicates the presence of populations of free and hydrogen-bonded C–H oscillators. The latter gives rise to the additional spectral intensity in the π band due to the ≡C–H bonds hydrogen-bonded to a single π-bond of nonpolar cyclohexene. This type of C–H hydrogen bond is reported here for the first time. It might not have been conceived before either because it is unexpected and is likely to be extremely weak. To elucidate the origin of the π band, we conducted a series of tests.

A free C­(*sp*)–H band is accompanied by a weak low-frequency band at ∼3290 cm^–1^ (Figures 1a, 2a) due to the Fermi resonance between the terminal C–H stretch fundamental and the combination of the first overtone of the terminal C–H bend and C≡C stretch fundamental. This Fermi band persists in a deuterated **MP** (Figure 1a) and other terminal alkynes.^24,47^ Since the free C–H band shifts to lower frequencies in CHXene, we tested whether the increased spectral intensity of the π band can be explained by perturbation of the Fermi resonance. In CCl_4_ and tetrachloroethylene, the free C–H band is at the same position as in CHXene (Figure 2b), making resonance condition in these solvents identical to the latter. Both display only minor linear intensity enhancements at the peak of the Fermi band, while its integral intensity remains essentially unchanged compared to the **MP** spectrum in CHX (Figure 2b, Figures S4–S5, Table S4). In contrast, in CHXene, the π band is significantly broader, and its integral intensity increases by >20-fold. Thus, we eliminate the Fermi resonance as a possible origin of the π band.

Alternatively, aggregation- and concentration-induced effects could play a role. At several hundred mM concentration, **MP** shows signs of aggregation in nonpolar solvents that manifest as a rise of the band at 3270 cm^–1^. This band is essentially identical with the C–H band of neat **MP** and overlaps with the π band in CHXene. However, this band disappears and no further spectral changes take place at or below 100 mM, indicating that monomers are obtained, and the aggregation is not operative. Detailed information about the concentration dependence in both CHX and CHXene is presented in Section S23. The spectrum in CHXene shown in Figure 2b is for low concentration (50 mM); thus, aggregation cannot explain the π band at <3300 cm^–1^ either.

Another mechanism could be envisioned where the π-stacking interaction between the π-systems of **MP** and CHXene is at the origin of this band. If exists, this interaction should be very weak (∼1 kcal/mol or less),^48^ as it would involve the interaction between a double and a triple bond (or the whole conjugated propiolate C≡C–C=O moiety), rather than between extended delocalized aromatic π-surfaces. To the best of our knowledge, no experimentally detectable interaction of this kind in liquid solution at room temperature has been reported. π-Stacking would have the strongest effect on directly involved C≡C/C=O bonds, while in contrast, we observe that compared to the >20 cm^–1^ effect on the C–H stretch observed in CHXene, the effect on both C≡C and C=O markers is ∼1 cm^–1^ (Tables S5–S6). Additionally, we compared the effect of changing CHX to CHXene for two control samples where a C­(*sp*)–H is substituted for a C­(*sp*)–CH_3_ and C­(*sp*)–Si­(CH_3_)_3_ with the rest of the **MP** molecule being intact. Such a modification dramatically increases the bulkiness of the substituents on the triple bond, which would disfavor π–π interactions. Nevertheless, the magnitude of the C≡C shift remains essentially the same (∼1 cm^–1^) among all 3 compounds (Tables S5–S6) and likely originates from the dispersion interaction with a polarizable double bond(s) in solvent as discussed in the following section.

We can also rule out any nonspecific field or dispersion interactions with the solvent, as those effects would only lead to shift and/or broadening of the existing free C–H band without splitting it into two.

We report an IR spectrum of **MP** in 1,3-cyclohexadiene that contains two conjugated double bonds (Figure 2c, CHX2ene). Compared to CHXene, the free C–H band is no longer distinguishable, with the π band gaining linear intensity by ∼2, while its position remains in place. This is fully consistent with H-bond formation, as the energy of the C–H···π HB is approximately constant in CHXene and CHX2ene, while the entropic contribution doubles due to the twice as large accessible π-surface area of the diene, thus favorably affecting the free energy of HB formation. The spectrum does not change when nonconjugated 1,4-cyclohexadiene containing two decoupled C=C double bonds is used (Figure 2c), supporting the entropic argument. Similarly, in esters and ethers when shortening the length of the alkyl chains, the H-bonded C–H band grows without shifting, while the free C–H peak decreases due to the entropic penalty (Figures S11–S12). Instead, π-stacking interaction would result in (i) larger energy stabilization of cyclohexadiene compared to cyclohexene, leading to a redshift of the C–H band and an even larger shift of the C≡C stretch that is not observed (Table S5), and (ii) stronger stabilization and hence the redshift of the C–H band in conjugated versus decoupled dienes, which is also not observed (Figure 2c).

To recap, we definitively rule out the Fermi resonance, nonspecific field effects, dispersion, aggregation-induced effects, and π-stacking as the origin of the π band in CHXene. Instead, we convincingly demonstrate that it arises from the weak but experimentally distinct C–H···C=C H-bond interaction, which we designate as a C–H-single π hydrogen bond to differentiate it from an HB to π-aromatic systems. To the best of our knowledge, this is the first demonstration of this type of interaction in room-temperature liquid between a nominally nonpolar C–H and a π-bond in a nonpolar hydrocarbon, which, according to conventional understanding of H-bonds, should not even exist. It illustrates the exceptional sensitivity of a C­(*sp*)–H group as a vibrational marker and an astounding difference that just two π-electrons can make to a truly nonpolar molecule featuring no polar bonds.

Further evidence of this elusive HB is provided by our NMR experiments. In stark contrast to aromatic solvents, in cyclohexene and cyclohexadiene, the δ^1^H of an alkyne proton engaged into C–H-single π HB is shifted downfield compared to saturated hydrocarbons (Figure 4a). It seems counterintuitive and contradicts the classical McConnell anisotropic cone model^49^ for alkenes, abundantly present in textbooks.^25^ The model predicts shielding and upfield shift for the protons above the double bond π-system similar to aromatic systems. However, the McConnell model only takes into account magnetic anisotropy and fails to account for steric and through-space van der Waals effects that within a short distance of <3 Å from the C=C plane dominate over the anisotropic term, as shown by modern calculations.^50–52^ Because the distance between the hydrogen-bond forming proton and the center of the σ-bond of the C=C is ∼2.5 Å, as evidenced by our quantum chemical calculations (Figure S9), ≡C–H is located in the deshielding region of the double bond. Our IR and NMR data are thus fully consistent with the HB formation and provide experimental validation for the theoretically suggested refined cone model.^50–52^

To explore the limits of the observed weak C–H···π hydrogen bonding, we investigated whether it applies to electron-poor double bonds. We measured the spectra of **MP** in alkenes with depleted π-electron density due to a different number of electron-withdrawing groups connected to the double bond: tetrachloroethylene, *trans*-dichloroethylene, and decafluorocyclohexene. No HB formation takes place with any of these alkenes similar to perfluorinated benzene in the aromatic series (Figure S10a). The spectrum in the least deactivated *trans*-dichloroethylene is identical to that in chloroform, which does not accept HBs (Figure S10b). The H-bond shift of only ∼20 cm^–1^ in CHXene indicates that this H-bond is probably at the boundary of what could be experimentally discerned for the C–H hydrogen bonding in liquid. By contrast, the interaction between π-systems of propiolate and the deactivated alkenes, if operative, would be favored as the molecular electrostatic surface potential around the triple bond of **MP** is negative, while these alkenes feature positive π-acidic surfaces (Figure S8) that could potentially lead to an even stronger π–π interaction with these solvents. No evidence of this interaction is observed.

### London Dispersion Dictates the Position of the Free C–H Peak

In solvents where the free C–H band is distinctly observed, its position varies. Figure 3a shows a few examples of the free C–H band that shifts by ∼25 cm^–1^. The shift does not seem to follow any intuitive behavior. In CS_2_, which is a nondipolar molecule containing polar bonds, the C–H band displays a large redshift. In CHXene with no polar bonds, it is slightly blue-shifted compared to CS_2_ but red-shifted versus dipolar di-isopropyl ether. In all these solvents, the free C–H band is red-shifted with respect to overall apolar but featuring many polarized C–F bonds perfluorohexane. The electric field effect described via the VSE cannot explain such variation.

**Figure 3:**
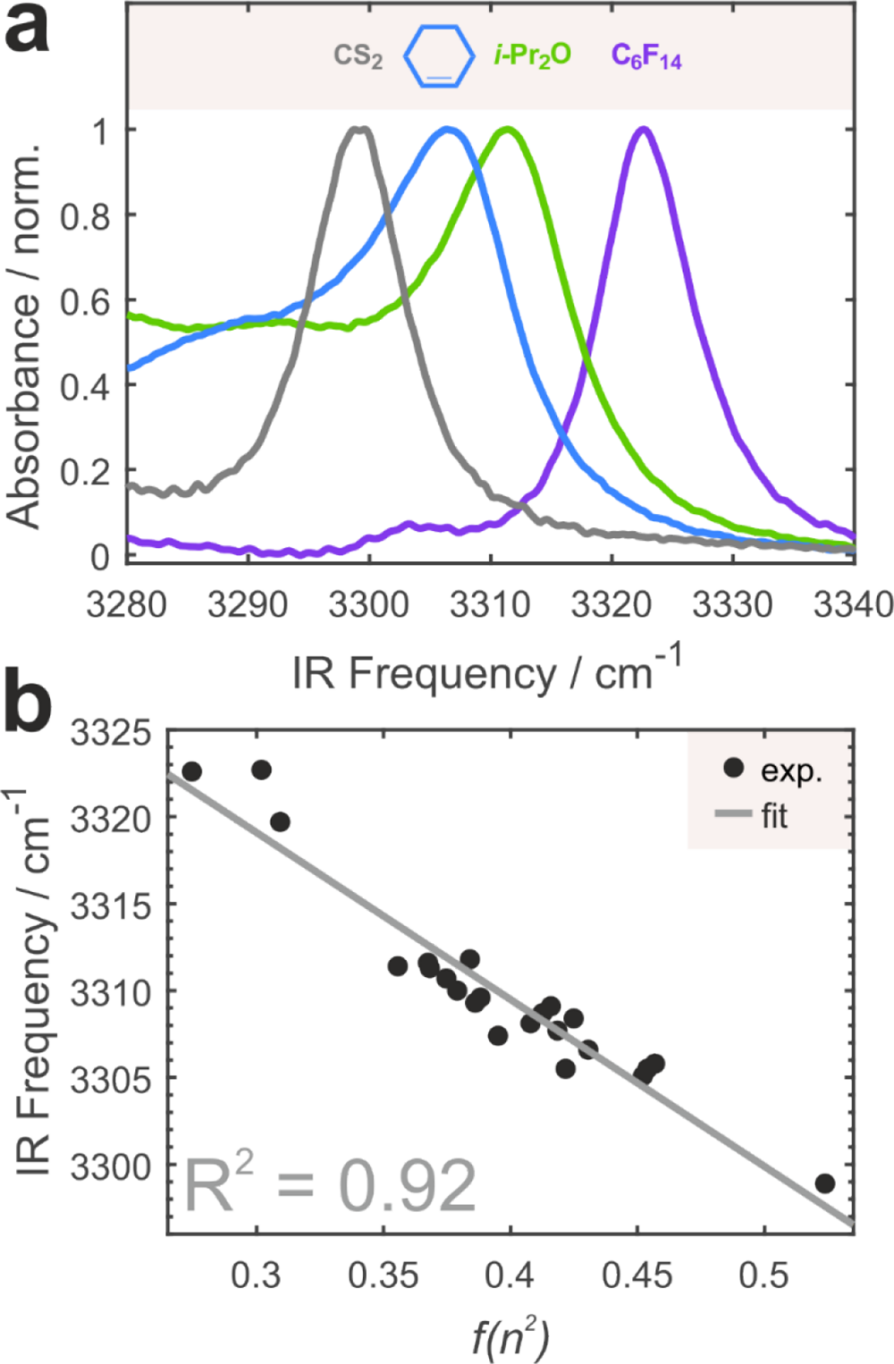
Free ≡C–H band as a sensor of London dispersion interactions. (a) IR spectra of the **MP** free ≡C–H stretch in several solvents of various nature. (b) ≡C–H stretch as a function of the Onsager electronic polarizability function *f*(*n* ^2^) as the simplest quantitative measure of dispersion. Experimental data points are shown as black markers, and linear fit is a gray line with its coefficient of determination shown.

Instead, it arises from attractive London dispersion interactions between **MP** and the respective solvents. Figure 3b shows that the frequency of the free C–H band is determined (*R* ^2^ = 0.92) by the Onsager electronic polarizability function *f*(*n* ^2^), which is the simplest physically meaningful metric of dispersion (Table S1): the larger the value of *f*(*n* ^2^), the more red-shifted is the free C–H band. This trend demonstrates that often underappreciated dispersion plays a significant role in shaping the spectrum. Recently, an interest has surged from both theoretical^53,54^ and experimental^55,56^ communities to understand how such an omnipresent nonspecific effect tailors chemical reactivity and can be harnessed in catalysis.^55,57,58^ Experimental methods to quantitatively evaluate dispersion in liquids remain scarce, especially for polar molecules such as **MP**, where other, stronger, intermolecular interactions often dominate observable properties.

In perfluorohexane and CS_2_, dispersion interactions dominate, and as evidenced by the single IR band, all C–H bonds of **MP** molecules experience similar environments. On the other hand, in ethers, we can differentiate the C–H bonds pointing to the oxygen atom of the solvent from those pointing to the hydrophobic alkyl tails. In the former, a distinct H-bonded IR band peaking at 3200–3220 cm^–1^ is observed as discussed above, while the latter ones are only stabilized by the weak dispersive interaction with the alkyl tails and appear at >3300 cm^–1^. Moreover, there should be an intermediate population, where a C–H points toward the oxygen atom of the ether but does not engage in H-bonding or is generally aligned with the solvent dipole. These frustrated configurations should result in a band significantly red-shifted compared to the free C–H band due to the favorable electric field effect as described by the VSE but blue-shifted compared to the H-bonded C–H. Such a band is indeed found in ethers as a shoulder at 3265–3275 cm^–1^ (Figure 1c). In the following section, we provide further justification for this statement using IR-NMR correlations.

### NMR Chemical Shifts of ≡C–H Are Excellent Reporters of the Local Electric Field

NMR has been used as an alternative to the VSE for sensing microscopic electric fields near the carbonyl^59,60^ and nitrile^61^ moieties. For C≡N, the combination of IR and NMR data yields an elegant method to decompose the vibrational frequency shift into the electrostatic term and the notorious nonelectrostatic hydrogen-bond blueshift.^62^ For C=O, H-bonding enhances the local field originating from nonspecific interactions.^63^ Inspired by these works, we wondered if the ≡C–H fragment can also serve in this role. For both C=O and C≡N, the ^13^C chemical shift is the only choice as an NMR marker in isotopically nonenriched molecules, as the most common isotopes of both N and O are NMR silent. In contrast, the ≡C–H moiety contains two NMR active nuclei, and we experimented with both δ^1^H and δ^13^C as electrostatic probes.

To obtain a reliable estimate of the electric field magnitude, including in the presence of strong hydrogen bonding, and to account for the charge transfer, polarization, and other nonelectrostatic components important for the ≡C–H···**A** hydrogen bond, we employed *ab initio* molecular dynamics (AIMD) simulations. They were performed for **MP** in diverse environments: cyclohexane, acetone, acetonitrile, di-*n*-butyl ether, THF, and DMSO. The electric fields projected onto the C­(*sp*)–H bond were calculated from AIMD trajectories (Figure S21). As shown in Figure 4b and Figures S21–S22, the C­(*sp*)–H bond senses a large range of microscopic fields: from a few MV/cm^–1^ in nonpolar cyclohexane to an intense field of 100 MV/cm^–1^ in DMSO where it is strongly H-bonded. Figure 4b and Figure S22 show that both δ^1^H and δ^13^C are nearly quantitatively linearly correlated with the electric field projection (*R* ^2^ > 0.90). Interestingly, the sensitivity parameter for the electric field – the NMR equivalent of the Stark tuning rate in IR spectroscopy – is ∼3.5 times larger for δ^13^C than for δ^1^H (Figure S22). The residual scatter likely comes from the difficulties in reaching the full convergence in 50 ps AIMD trajectories due to the high computational cost of simulations. One could certainly use classical MD simulations to achieve better statistical convergence. We do, however, emphasize the importance of quantum-mechanical treatment for simulating H-bonding in systems of interest here. Alternatively, machine-learned potentials are becoming increasingly popular in condensed-phase simulations.^64–69^

**Figure 4:**
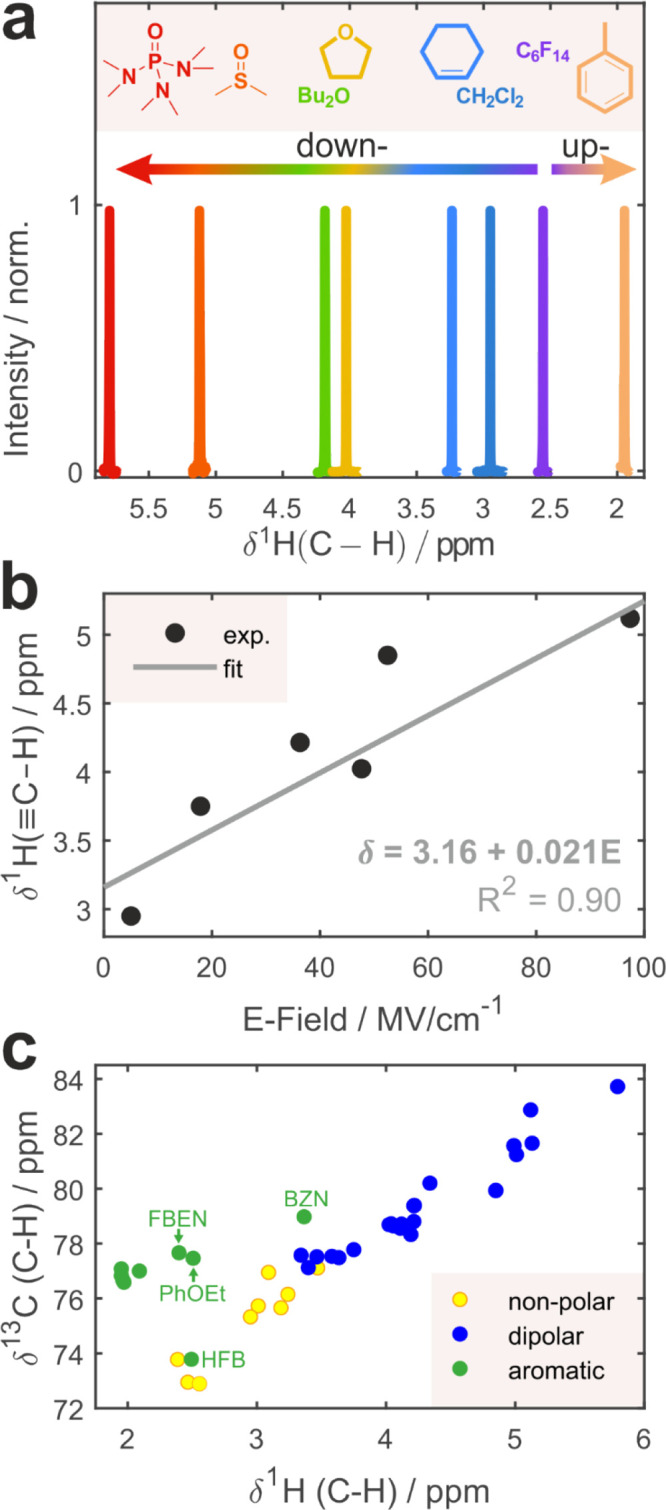
NMR spectroscopy. (a) ^1^H NMR spectra of **MP** alkynyl ≡C–H in several representative solvents, whose structures are shown. (b) Experimental ^1^H NMR chemical shift of alkynyl ≡C–H versus simulated electric fields projected onto the C–H bond. Experimental data points are shown as markers; linear fit with its respective coefficient of determination and fit equation is indicated. (c) Correlation between ^1^H and ^13^C chemical shifts across a broad range of nonpolar (yellow), dipolar (blue), and aromatic (green) solvents. Several notably dipolar aromatic solvents are indicated (FBEN, fluorobenzene; PhOEt, phenetole; BZN, benzonitrile). HFB, hexafluorobenzene, is an aromatic analogue of nonpolar solvents.

Figure 4c shows that ^1^H and ^13^C chemical shifts are strongly linearly correlated over the whole range of nonpolar and dipolar solvents, regardless of whether they are H-bonded or not. However, the dependence is nonmonotonic when aromatic solvents are included due to the unique upfield shift that the proton experiences upon aromatic H-bonding, while ^13^C is not shielded by the aromatic π-electron density, because it is more distant, which explains the characteristic V-shaped dependence of δ^13^C on δ^1^H. The trend holds consistent across the series of dipolar solvents containing aromatic fragments (fluorobenzene, phenetole, benzonitrile). They could be classified into both dipolar and aromatic categories, and we highlight them in Figure 4c. If they did not contain an aromatic π-system, their δ^1^H would appear downfield in accord with their polarity, but the π-system provides an alternative competing C–H···π interaction, and since NMR measures the ensemble average across all possible configurations weighted by their respective populations, the presence of this π-bonded population leads to lower δ^1^H than in the respective nonaromatic dipolar solvent. While, in both aromatic and nonaromatic categories, the enhancement of the field and/or H-bond accepting ability leads to the correlated increase in ^1^H and ^13^C shifts, it can be viewed that aromatic solvents form a separate linear trend offset to lower δ^1^H and higher δ^13^C values compared to nonaromatic ones.

### Dissecting Microscopic Intermolecular Interactions Based on Purely Experimental Data

The results presented above indicate that the NMR chemical shifts of both atoms in the C­(*sp*)–H bond can be used to sense the microscopic electric fields. This is in stark contrast to the C­(*sp*)–H IR frequency shift that is sensitive to the sum of electrostatic interactions and induction (charge transfer and polarization).^24^ As such, we combine the IR and NMR data to produce IR-NMR correlations, which allow us to distinguish and quantify the electric field effect versus nonelectrostatic contributions based on the experimental data alone. Figure 5 shows such a correlation between the δ^1^H and ≡C–H stretch frequency. Three distinct regions are observed (Figure 5a):

i. an “electrostatic line”: a nearly quantitative (*R* ^2^ > 0.98) linear dependence that encompasses environments ranging from nonpolar through weakly polar to strongly polar but all non- to weakly H-bonding (e.g., acetonitrile and acetone). This line is produced by fitting 13 diverse systems and quantifies the electric field effect in the range of solvents where H-bonding is either nonexistent or weak enough such that it reduces to an electric field effect with negligible induction. This dependence is equivalent to the VSE. The frustrated band observed in 5 different ethers at 3265–3275 cm^–1^ and ascribed to the C–H configuration pointing to the oxygen but not hydrogen-bonded to it (Figure 1c, Figure S14) falls precisely on this electrostatic line (magenta markers, Figure 5b), further reinforcing this interpretation.
ii. hydrogen-bonding in dipolar solvents where the nonelectrostatic component is sizable (Figure 5a, blue triangular region): the IR shift is significantly larger than the prediction from the magnitude of the local electric field. This leads to the vertical downward deviation from the electrostatic line. The magnitude of this extra shift of IR frequency quantifies the nonelectrostatic effects such as polarization, charge-transfer, and others. The deviations from the electrostatic trend increase in more strongly HB accepting solvents conferring a triangular shape to this region. This agrees with a known H-bond behavior, as a stronger HB means both stronger electrostatic interaction between the ≡C–H and accepting fragment and higher covalency originating from nonelectrostatic interactions. The observed amount of nonelectrostatic contribution is in accordance with Pearson’s hard and soft acids and bases (HSAB) principle^70,71^ according to which a soft ≡C–H acid interacts stronger with soft bases, resulting in a larger covalent component of this interaction. Ketones and nitriles are the hardest bases, and both acetone and acetonitrile fall onto the electrostatic line as the nonelectrostatic contribution is minimal. Esters are softer than ketones, and they demonstrate the smallest vertical shift from the electrostatic line, while ethers are soft bases bringing about a much larger nonelectrostatic contribution and thus shifted further downward. On the other hand, sulfides are even softer than ethers and interaction with them leads to larger relative induction despite the weaker overall H-bond accepting ability of this solvent class (Figure 5b, S13). The amount of the nonelectrostatic shift extracted in this way correlates with the Gutmann donor number (Figure S19), with the same (within ∼10%) sensitivity parameter as previously reported by Boxer et al.,^24^ where the classical MD simulations were used to obtain the electric fields and produce an electrostatic line. Here, we demonstrate that combination of two common experimental techniques can be used to construct such electrostatic calibration, obviating the need for simulations.
iii. Aromatic solvents break the monotonic dependency and form a separate trend with the opposite sign of the slope. This trend is clearly observed with systems where the aromatic π-system is the only HB acceptor: they are located in the leftmost part of the green area in Figure 5a. Interestingly, the dipolar solvents containing an aromatic moiety (such as phenetole, PhOEt, and benzonitrile, BZN) do not follow either trend. This is because there are two competing factors at play. The dipolar interactions with the ether or nitrile part of the molecule determine their positioning in the right (dipolar) part of the nonmonotonic dependence, while the competing HB interactions with the aromatic moiety tend to shift the δ^1^H upfield to the left part of the plot (Figure 5a). As such, they appear more (phenetole) or less (benzonitrile) shifted from the dipolar trend (Figure 5b) as the field effect is weaker or stronger, and the π-system is activated or deactivated toward hydrogen bond acceptance correspondingly.

**Figure 5:**
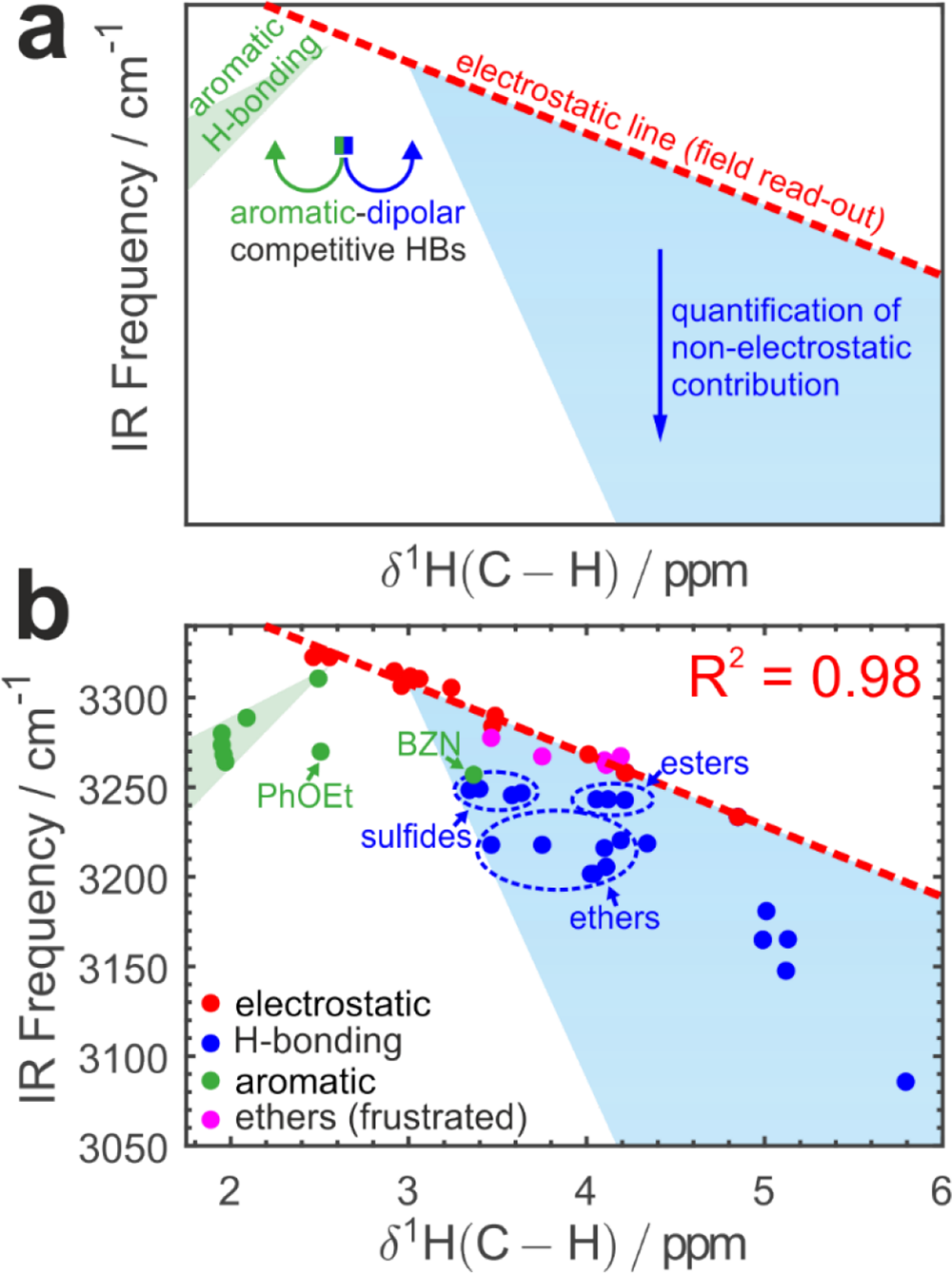
Disentangling and quantifying electrostatics and short-range effects. (a) General concept of ≡C–H IR-NMR correlations and cartography of accessible regions. Vertical deviation from the electrostatic line (blue region) quantifies the amount of nonelectrostatic contributions. (b) Experimental δ^1^H NMR and ≡C–H stretch IR data for **MP**. Electrostatic data points shown in red yield the electrostatic line (coefficient of determination indicated), blue markers correspond to strongly H-bonded systems (several chemical classes indicated), and magenta markers show the independently extracted frustrated band in ethers and fall onto the electrostatic line. Green markers denote aromatic systems. Benzonitrile (BZN) and phenetole (PhOEt), where two types of HB interactions (≡C–H··· π vs ≡C–H···**A**) compete, are labeled explicitly.

Combining the electric field dependence of the NMR ^1^H chemical shift (Figure 4b) and IR-NMR electrostatic line correlation (Figure 5b), we obtain the Stark tuning rate of 0.83 cm^–1^/(MV/cm^–1^) for the ≡C–H stretch. This value is 2–3 times larger than that for workhorse Stark probes of C=O/C≡N.^26,35,72^ Perhaps a more important utility of this marker comes from even larger sensitivity to the nonelectrostatic components of the H-bond interaction and to London dispersion interactions when non-H-bonded ≡C–H is concerned. The approach of using combined IR and NMR data allows us to quantitatively decompose the various contributors to the IR shift. When only one distinct C–H population is present, it is not clear how one can distinguish weak H-bonding from non-H-bonding but strongly field-stabilizing environments where VSE leads to the redshift of the C–H transition. For example, in bromobenzene or phenetole from the IR spectrum alone, it is impossible to infer whether H-bonding to the π-system is operative, and given the trend (Figure 2a), it is tempting to conclude that no H-bonding occurs. In contrast, from the IR-NMR correlation (Figure 5b), H-bond formation is evident.

To validate our experimental decomposition of H-bond interactions into electrostatic and nonelectrostatic terms, we calculated the H-bond energies and their physical components for the two H-bond complexes of **MP** with ethyl acetate and THF using the gold standard Symmetry-Adapted Perturbation Theory, SAPT2+(3)­δMP2 (Section S1.4.5, Table S8).^73^ As predicted by our decomposition in Figure 5b, while the electrostatic contributions are nearly identical for both these solvents (−6.2 vs −6.5 kcal/mol, respectively), the nonelectrostatic components, namely, induction (−1.8 vs −2.0 kcal/mol), exchange (5.8 vs 7.2 kcal/mol) and dispersion (−2.6 vs −3.2 kcal/mol) components vary significantly with THF having a larger nonelectrostatic contribution for all of them, in line with both the decomposition and Pearson’s HSAB principle.

### Direct Observation of the Hydrogen Bond Formation in THz Spectroscopy

So far, we have focused on sensitive and highly informative observations of indirect metrics of H-bonding using changes in spectroscopic properties of the donor ≡C–H fragment to establish its existence. On the other hand, H-bond formation is expected to produce the two new modes: the H-bond stretch and bend low-frequency vibrations in the far-infrared, or terahertz (THz), range. Detecting these modes is the most direct observation of the hydrogen bond from the spectroscopic perspective.^74–76^ To investigate this spectral region, we performed time-domain THz spectroscopy measurements using subpicosecond broadband (0–6 THz) pulses obtained in a custom-built setup (Section S1.4.3).^77^ This unique spectrometer based on a high repetition rate ultrafast laser utilizes rapid-scan multichannel detection^77^ with a small-bias electro-optic sampling scheme^78,79^ enhanced by B-matrix referencing^80^ to enable fast and efficient measurements of highly volatile samples. It allowed us to comprehensively probe H-bond formation across a wide selection of ≡C–H H-bonding partners.

Figure 6a shows the THz interaction spectra obtained by subtracting the spectra of neat components, weighted by their respective molar fractions, from the spectrum of the **MP** solutions in dipolar solvents. The interaction spectrum represents the deviation from the additive ideal solution behavior due to interactions between the two components. Two intense bands appear in the 0.3–5.5 THz (10–180 cm^–1^) region: the stronger higher-frequency one at the 3.5–6 THz region (115–180 cm^–1^) and a weaker band at 1.5–4 THz (50–130 cm^–1^).

**Figure 6:**
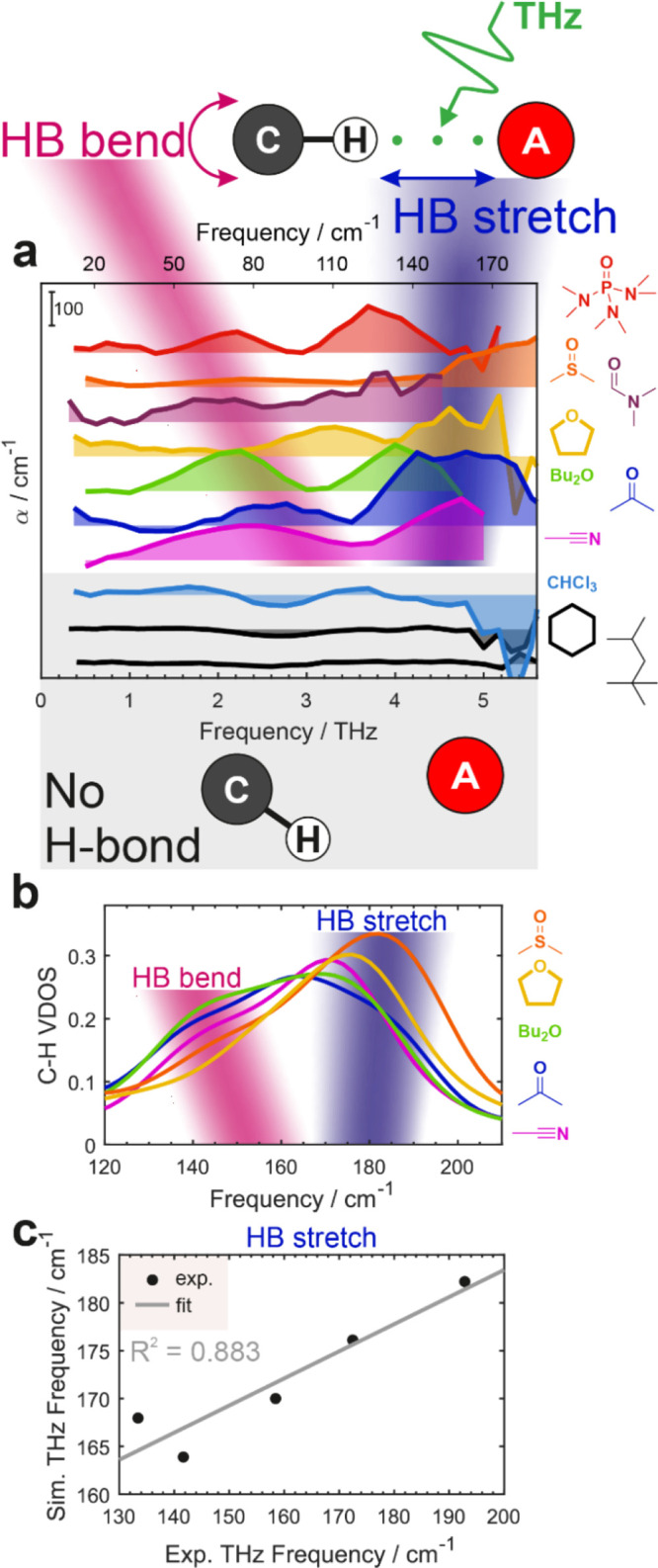
Direct observation of a ≡C–H···**A** hydrogen bond with broadband THz spectroscopy. (a) Experimental THz interaction spectra of **MP** in various solvents. The schematic at the top shows the simplified representation of the HB stretch and bend modes. Three control systems with no HB formation are grayed out at the bottom. (b) VDOS of the alkynyl proton in the experimentally relevant frequency range calculated using *ab initio* MD simulations. (c) Correspondence between the maxima of the experimentally observed and simulated HB stretch band. Linear fit along with the coefficient of determination is shown.

In contrast, no new bands appear in solvents that cannot participate in H-bonding with **MP** (hydrocarbons: cyclohexane, isooctane) or that can only act as H-bond donors, but not acceptors (CHCl_3_). There are either no changes in these solvents (hydrocarbons), or their magnitude is much smaller and the shape is qualitatively different (chloroform). Therefore, we assign the high-frequency band to the H-bond stretch and the low-frequency band to the H-bond bend. Qualitatively, these bands resemble the THz spectrum of liquid H_2_O, which exhibits the ∼6 THz and ∼2 THz bands, corresponding to the HB stretch and bend modes, respectively.^41,81^

Further support for this interpretation comes from our AIMD simulations. Specifically, we computed the vibrational density of states (VDOS) using the C­(*sp*)–H proton velocity autocorrelation function as explained in Section S1.4.4. In the experimentally relevant THz region at 120–210 cm^–1^, a band with a low-frequency shoulder appears (Figure 6b). This band is associated with the H-bond stretch mode, while the shoulder on the low-frequency side comes from the H-bond bend. The absolute frequency of the H-bond stretching band is slightly red-shifted compared to the experiment, rendering the bending mode not as well-resolved, but the trend with the solvents is in excellent agreement with the experimental data (Figure 6c). Simulations confirm that the nature of these modes is associated with the intermolecular alkynyl C–H hydrogen bonding collective modes.

The experimental THz bands shift as the H-bond accepting ability of the solvent increases: the low-frequency bend continuously red-shifts, while the stretching mode blueshifts. The direction of these shifts is in line with the expected trends upon H-bond strengthening: the longitudinal HB mode stiffens, while the transverse HB mode softens. The shifts in this region are the opposite of the commonly observed trends in the mid-IR for the H-bond donor X–H stretch and bend modes. Further details on THz analyses are presented in Section S2.8.

### ≡C–H···A Hydrogen Bonds Satisfy Geometric Criteria for Typical Strong Proper Hydrogen Bonds

The good agreement between simulations and experiment encouraged us to use simulations to analyze the structural parameters of C–H···**A** hydrogen bonds. Figure 7a,b shows two examples of 2D spatial distribution functions for the C···**A** distance (Figure 7a) and H···**A** distance (Figure 7b), where **A** is the H-bond acceptor atom, versus C–H···**A** angle. Complete data are shown in Figures S23–S24. Clearly, in all HB accepting solvents, there is a strong preference for an H-bond formation – a strong peak at 3.05–3.35 Å and a linear 180° arrangement dominate the C···**A** distributions. The H···**A** distance peaks at 1.95–2.35 Å, which is significantly shorter than the sum of the van der Waals radii of H and O atoms of 2.7–2.8 Å (Figure 7b).^82^ In contrast, no such peak or, to that end, any preferential spatial arrangement exists in cyclohexane.

**Figure 7:**
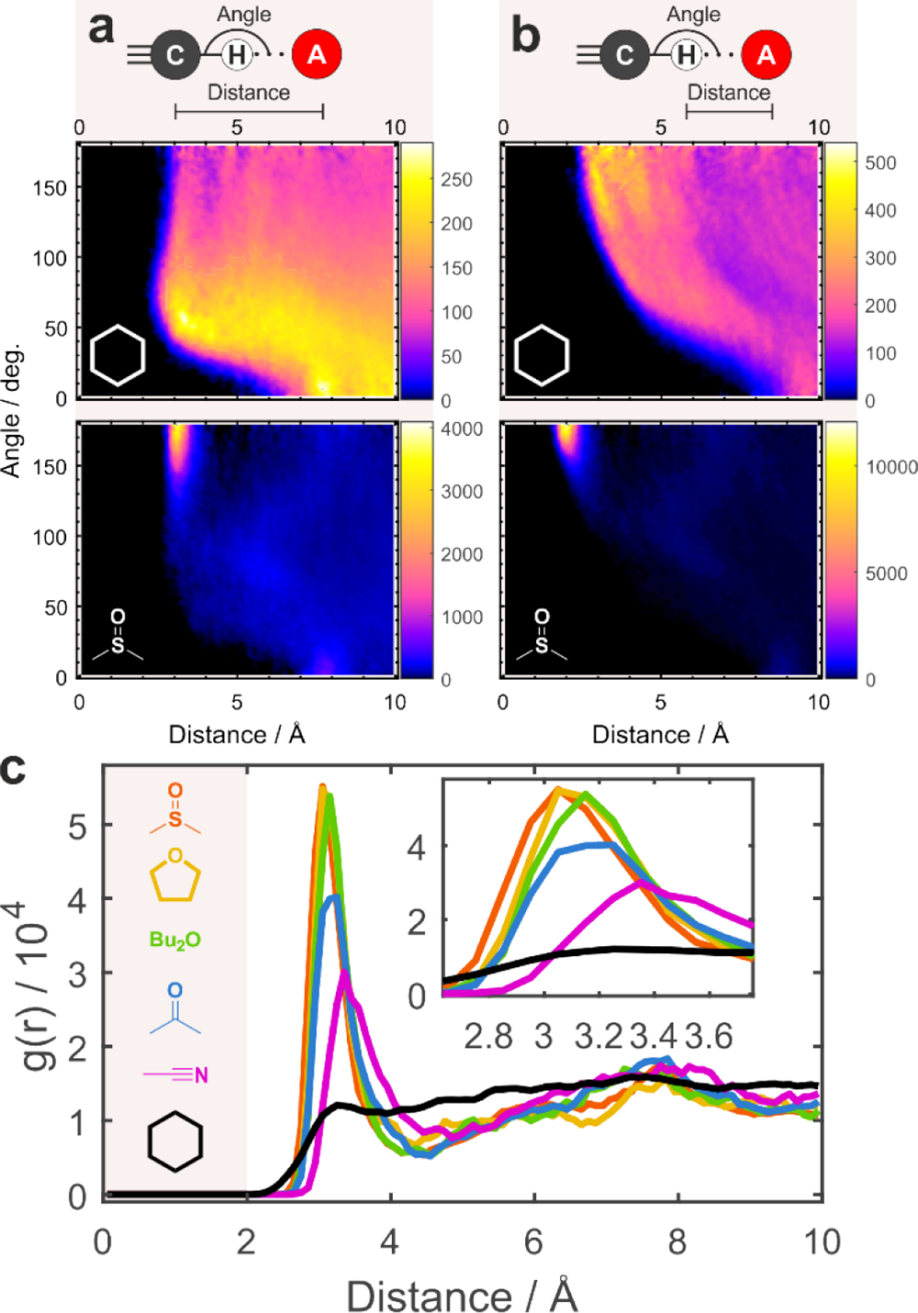
≡C–H···**A** hydrogen bond distributions extracted from AIMD simulations in strongly H-bonding DMSO (bottom) versus non-H-bonding CHX (top). (a) 2D spatial distribution function of the C···**A** distance versus C–H···**A** angle. (b) 2D spatial distribution function of H···**A** distance versus C–H···**A** angle. (c) Radial distribution function of the C···**A** distance for all simulated solvents obtained via integration of the spatial distribution function over the angular coordinate. The inset zooms into the first peak, which shifts to shorter distances, narrows, and grows as the H-bond accepting ability of solvents increases.

Integrating over the angular coordinate, we obtain the radial distribution function shown in Figure 7c, whose first peak corresponds to the H-bond heavy-atom distance between C and **A**. This peak grows, narrows, and shifts (by >0.3 Å) to smaller distances as the H-bond accepting ability of the solvent increases, as the free-energy well of the hydrogen-bonded C–H progressively deepens. We note that **MP** forms significantly shorter and thus stronger HBs compared to those of 1-hexyne^24^ as revealed by narrower and shifted maximum of the distribution in strongly H-bond accepting DMSO by ∼0.5 Å. Additionally, we do not observe any strong correlation between the C···**A** distance and the angle reported previously.^24^ These computational data reinforce our interpretation of the strong proper hydrogen bond formation between alkynyl C–H and solvents.

## Conclusions

We comprehensively investigated a novel, previously underappreciated alkynyl C­(*sp*)–H local vibrational marker. It is a compact, readily incorporable diatomic local mode distinct from all other C–H stretches in organic molecules. We observed an impressive and not previously reported 237 cm^–1^ span of **MP** ≡C–H stretch peak absorption frequencies upon systematically varying its environment across >50 media. Strong proper C–H hydrogen bonding is a dominant source of this spectral variation. The C–H hydrogen bonding is operative not only to electronegative heteroatoms, such as oxygen and nitrogen, but also to aromatic π-systems and even to single π-bonds in nonpolar hydrocarbons. Observation of similar effects in **TMS–CCH** indicates the general nature of the C­(*sp*)–H hydrogen bonding regardless of the presence of the EWG attached to the alkyne.

This strong hydrogen bonding ability is anticipated to play a role in endowing terminal alkyne containing molecules with structure and function. For example, bottlebrush polymers designed for “grafting onto” click reactions often feature terminal alkynes as side chains^83,84^ and might be envisioned to adopt unique secondary and tertiary structures, similar to how conventional O–H and N–H H-bonds dictate structures of (bio)­polymers. It is also expected to be an important factor facilitating molecular recognition and enhancing the reactivity of terminal alkynes, for example, in the alkyne–azide click reaction. Some of us have previously reported how relatively weak excited-state halogen bond formation provides orientational constraint that enables ensuing bimolecular electron transfer.^85^ We imagine that a strong hydrogen bond can play this role even more effectively and be relevant for excited-state proton transfer reactions.

Spontaneous H/D exchange in neutral **MP** solution takes place within seconds and indicates the labile nature of its C–H bond, like conventional O–H and N–H H-bond donors. Despite its similarity to O–H, ≡C–H features unique advantages compared to the latter. First, the proton in ≡C–H can be made either exchangeable or nonexchangeable by tweaking the substitution at the alkyne. Second, unlike the O–H group that simultaneously acts as a hydrogen bond donor and acceptor, ≡C–H does not form H-bonding networks and remains a highly localized and decoupled vibrational mode as it is only a hydrogen bond donor but not acceptor.

Using broadband THz spectroscopy, we directly observe the low-frequency stretching and bending vibrations of the forming H-bond, providing a unique window into the structural dynamics of proper C–H hydrogen bonding. Beyond simply confirming the presence of hydrogen bonds, these measurements demonstrate the strength of the interaction, offering insight into how subtle changes in the H-bond accepting ability and surrounding polarity influence the THz spectral characteristics of this interaction and the collective dynamics of surrounding H-bonding partners. Experimental assignments are validated with AIMD simulations that are also used to elucidate the structural characteristics and distribution of the H-bond parameters in liquids. AIMD confirms preference for short contacts and linear arrangement between the *sp* carbon and the H-bond acceptor.

Using an array of structure-sensitive techniques, such as mid-IR and ^1^H and ^13^C NMR spectroscopies, we go beyond merely detecting the presence of H-bonds. Instead, we lay out a simple and robust experimental approach validated by simulations on how to decompose the observable H-bond-induced IR frequency shifts into the electric field effects and short-range nonelectrostatic contributions. Until now, it was not possible without high-level simulations that could faithfully reproduce H-bonding and its interaction components.^24^ The idea of the electric field as a new catalytic agent for nonredox reactions that underpins the extraordinary catalytic proficiency of enzymes has been gaining traction.^27,86–89^ Carbonyl or nitrile modes are frequently used to sense the field at the active site, and this work demonstrates that easily incorporable terminal alkynes^90–92^ will be useful for simultaneous probing of field and nonelectrostatic contributions in the future. Beyond biocatalysis, it has been suggested that the electric field can serve as a “smart reagent” in chemical reactions,^93^ including industrial ones,^94,95^ opening a new catalytic modality for many practical transformations.^93–96^ But the unambiguous separation of the electric field effect from the myriad of other factors influencing reactivity remains a formidable task. Tools allowing for separation of these effects to systematically map catalytic mechanisms will prove immensely useful for understanding the full potential of this novel approach. An easily installable terminal alkyne moiety is one example of such a marker, as we show in this work.

Furthermore, for non-H-bonded C­(*sp*)–H, we identify dispersion and electrostatic contributions as important factors determining its stretch frequency. It allows us to systematically track the London dispersion component experimentally and evaluate it in complex systems that are not composed of only hydrocarbons but contain various polar moieties. London dispersion has recently emerged as an attractive design element influencing reactivity in sterically congested molecules.^57,58^ Its experimental evaluation remains challenging and can often be inferred only indirectly. An easily installed terminal alkyne marker may prove useful in alleviating this difficulty.

Overall, decomposition of the observed IR frequency shifts into physically meaningful components is crucial for exploiting this vibrational mode in advanced applications. For example, IR frequencies are being increasingly used as predictors in machine learning approaches to predict reactivity and understand reaction mechanisms.^97,98^

## Supplementary Material



## Data Availability

The data reported in this article have been deposited to Zenodo and can be accessed at the following DOI:10.5281/zenodo.19816058.

## Notes

Experimental and computational details and methods, synthesis of deuterated methyl propiolate, safety information, additional spectroscopic data and details, additional simulations data and details, Figures S1–S27, Tables S1–S8 (PDF)

## Notes

Dedicated to Prof. Peter Hamm on the occasion of his 60th birthday.
