# Supplementary material for: Strong Proper C–H Hydrogen Bonds: Experimental Evidence across Spectral Ranges
Source: JACS Au. 2026 May 5;6(5):2988–3001. doi: 10.1021/jacsau.6c00326 (PMC13213511; doi:10.1021/jacsau.6c00326)
Supplement: Supplementary file 1 [file au6c00326_si_001.pdf]

SUPPORTING INFORMATION

Strong Proper C-H Hydrogen Bonds: Experimental Evidence across Spectral Ranges

Anton Kliuchynskyi,[1] Aritri Biswas,[2] Andrey Shalit,[1] Alexei A. Kananenka,[2] Bogdan Dereka[1*]

[1]*Department of Chemistry, University of Zurich, CH-8057 Zurich, Switzerland*

[2]*Department of Physics and Astronomy, University of Delaware, Newark, Delaware 19716, USA*

* Corresponding author, e-mail: bogdan.dereka@chem.uzh.ch

Table of Contents

S1 EXPERIMENTAL DETAILS ... ***S2***

S1.1 Materials ... **S2**

S1.2 Safety and Hazards Information ... **S3**

S1.3 Deuterated Methyl Propiolate: Proton Exchange for Deuterium ... **S4**

S1.4 Methods ... **S5**

S1.4.1 Steady-State IR Spectroscopy ... S5

S1.4.2 NMR Spectroscopy ... S5

S1.4.3 Terahertz (THz) Spectroscopy ... S6

S1.4.4 Molecular Dynamics Simulations ... S6

S1.4.5 Quantum Chemical Calculations ... S8

S2 ADDITIONAL SPECTROSCOPIC DATA AND DETAILS ... ***S9***

S2.1 Calculated Structures and Frequencies of H-bonded MP ... S9

S2.2 Fermi Resonance ... S10

S2.3 Concentration Dependence and Aggregation Effects ... S12

S2.4 π-π Interactions ... S14

S2.5 Free and H-bonded C–H ... S17

S2.6 Non-Electrostatic Contribution vs. Gutmann Donor Number ... S23

S3 ADDITIONAL SIMULATIONS DATA AND DETAILS ... ***S27***

S4 SUPPLEMENTARY REFERENCES ... ***S34***

S1 EXPERIMENTAL DETAILS

S1.1 Materials

Methyl propiolate (**MP**) (99% purity) was bought from both Sigma-Aldrich and abcr Swiss AG, stored in a fridge under inert atmosphere and used as received. If stored properly, no yellow color develops over time. No difference was observed between the samples obtained from the two vendors. Samples were prepared by dissolving methyl propiolate in solvents listed in **Table S1** to reach the required concentration. Fresh samples were prepared every time before the measurements and have not been reused. For the fast-reacting samples (TMU and TMG solvents are highlighted in red as they react with methyl propiolate), the effort has been made to perform the steady-state IR and NMR measurements as rapidly as possible after preparing the sample to ensure the sample integrity. TMG reacts too fast such that these efforts did not succeed, and no reliable spectra were measured in this solvent. All solvents were of spectroscopic grade. Hygroscopic solvents were stored and handled in the nitrogen-filled glovebox (MBraun LABmaster Pro).

Trimethylsilylacetylene (**TMS–CCH**) was bought from Sigma-Aldrich and Chemie Brunschwig and used as received. No difference was observed between the samples obtained from the two vendors. Fresh samples were prepared every time before the measurements and have not been reused.

Table S1. Solvents used in the current study to prepare solutions of methyl propiolate and **TMS–CCH** (n denotes refractive index, ε is a dielectric constant, $f(n^2)$ is the Onsager electronic polarizability function calculated as $f(n^2) = 2(n^2 - 1)/(2n^2 + 1)$.

No.	Solvent	Acronym	$\boldsymbol{n}$	$\boldsymbol{\varepsilon}$	$\boldsymbol{f(n^2)}$
1	Perfluorohexane	perFHex	1.2520	1.57	0.2745
2	Perfluorooctane	perFOct	1.2840	1.8100	0.3019
3	Decafluorocyclohexene	DFCHXene	1.2930	N/A	0.3093
4	2-Methylbutane	MeBu	1.3540	1.83	0.3571
5	Isooctane	IOCT	1.391	1.94	0.384
6	Cyclohexane	CHX	1.426	2.02	0.4079
7	Cyclohexene	CHXene	1.4470	2.2200	0.4217
8	1,4-Cyclohexadiene	CHX2ene-decouple	1.473	2.26	0.4381
9	1,3-Cyclohexadiene	CHX2ene-conj.	1.4740	2.6600	0.4388
10	Tetrachloroethylene	TCE	1.5040	2.5000	0.4569
11	*Trans*-dichloroethylene	DCE	1.4440	2.2700	0.4198
12	Carbon disulfide	CS2	1.6280	2.6400	0.5239
13	Hexafluorobenzene	HFB	1.3780	2.0500	0.3747
14	Fluorobenzene	FBEN	1.4660	5.5500	0.4338
15	Bromobenzene	BrBEN	1.5580	5.1700	0.4876
16	Benzene	BEN	1.5010	2.2800	0.4551
17	Toluene	TOL	1.4970	2.3800	0.4528
18	p-Xylene	pXYL	1.4960	2.3000	0.4522
19	Mesitylene	MES	1.4990	2.4000	0.4539
20	Phenyl ethyl ether (phenetole)	PhOEt	1.5070	4.5	0.4587
21	Di-n-octyl ether	DOE	1.4330	N/A	0.4126
22	Di-n-butyl ether	DBE	1.3970	3.10	0.3882
23	Tert-butyl methyl ether	MTBE	1.3690	4.5	0.3682
24	Di-isopropyl ether	DiPrE	1.3680	4.04	0.3675

25	Diethyl ether	DEE	1.3520	4.3300	0.3556
26	Di-n-butyl sulfide	DBS	1.4520	4.4100	0.4249
27	Di-isopropyl sulfide	DiPrS	1.4380	5.81	0.4159
28	Diethyl sulfide	DES	1.4420	7.2000	0.4185
29	Carbon tetrachloride	CCl4	1.4610	2.2400	0.4306
30	Chloroform	CHCl3	1.4460	4.81	0.4211
31	Dichloromethane	DCM	1.4240	8.93	0.4066
32	Tetrahydrothiophene	THT	1.5030	8.61	0.4563
33	Tetrahydrofuran	THF	1.4070	7.58	0.3951
34	Tetrahydrofuran-d_8	THF-d_8	1.4070	7.5800	0.3951
35	Butyl acetate	BuAc	1.3940	5.1	0.3861
36	Propyl acetate	PrAc	1.3840	6.3	0.3790
37	Ethyl acetate	EtAc	1.3720	6.4000	0.3704
38	Benzonitrile	BZN	1.5280	26.0	0.4709
39	Dimethyl acetamide	DMA	1.4380	37.7800	0.4159
40	Dimethylformamide	DMF	1.4310	36.71	0.4112
41	Dimethylcyanamide	DMCA	1.4100	37.2300	0.3971
42	Acetone	AC	1.3590	21.4	0.3609
43	Chloroacetonitrile	ClACN	1.4240	30.0000	0.4066
44	Acetonitrile	ACN	1.3440	38.8	0.3496
45	Dimethyl sulfoxide	DMSO	1.4790	46.68	0.4418
46	Hexamethylphosphoramide	HMPA	1.4660	30	0.4338
47	Methyl propiolate (neat)	PEster	1.4080	N/A	0.3958
48	Tetramethylurea	TMU	1.4500	24.46	0.4236
49	1,1,3,3-Tetramethylguanidine	TMG	1.468	23.10	0.4350

S1.2 Safety and Hazards Information

As safety is paramount in experimental chemical science,[S1] we would like to draw special attention to the hazards of methyl propiolate that we have experienced firsthand. Its safety data sheet[S2] indicates that methyl propiolate is a mildly irritating chemical for skin and eyes (OSHA Category 2). We stress that some of us have developed a strong reaction to it over time, even while taking extra safety precautions. Its vapor has a potent lacrimating ability that causes strong and persistent eye irritation. Most importantly, this chemical may cause severe and lasting skin burns even without direct skin contact but through two layers of nitrile gloves. Upon glove contact with the chemical, the skin damage develops slowly over the course of several days starting as eczema-like symptoms that manifest as burning, itching and swelling that gradually increase in intensity. The damage tends to spread beyond the area of contact. **Fig. S1** shows the two fingers affected by a small volume (100-200 μL) of concentrated methyl propiolate solution upon sample cell preparation for terahertz measurements through the two layers of gloves at 5 days after the incident. Dramatic swelling and rash are noticeable well beyond the area of exposure. Some skin areas slowly started healing, while others were still experiencing severe inflammation and swelling. After the peak damage is reached, it takes a couple of weeks before the skin regenerates and up to a month for the full recovery.

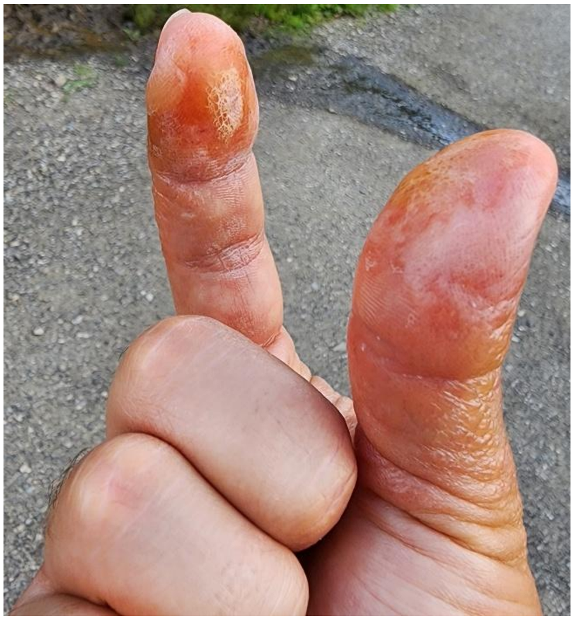

Figure S1. Photo of a thumb and an index fingers affected through two glove layers by a small squeezed out volume of concentrated methyl propiolate solution during the sample cell preparation. The picture was taken 5 days after the incident.

While we noticed that this might be an individual reaction that only some of us have experienced, we urge practitioners to take extra precautions upon dealing with this compound. It was also the case that each subsequent contact has caused more severe consequences despite increasing levels of precautions. We strongly recommend using nitrile gloves on top of the thick chemical resistant gloves when handling this compound and thoroughly rinse them immediately upon spilling even the smallest droplets of methyl propiolate on them.

S1.3 Deuterated Methyl Propiolate: Proton Exchange for Deuterium

Methyl Propiolate-d (MP-d). A 100 mL round-bottom flask was charged with methyl propiolate (5 mL) and D_2O (50 ml), filled with argon, closed with a glass lid and stirred vigorously overnight. After that, the resulting biphasic mixture was extracted with small amount of DCM (5×3 mL), organic phase was added to fresh D_2O and stirred overnight. The procedure was then repeated once more, for a total of 3 rounds of exchange. Organic phase was carefully distilled under mild vacuum through Vigreux column filled with glass beads to separate DCM from deuterated methyl propiolate (bp ~50 C° at 200 hPa). Deuteration reached 99.5%. ^{1}H NMR (400 MHz, $CDCl_3$) δ 3.80 (s, 3H). Chemical shifts (*δ* ppm) were determined with deuterochloroform as the internal reference.

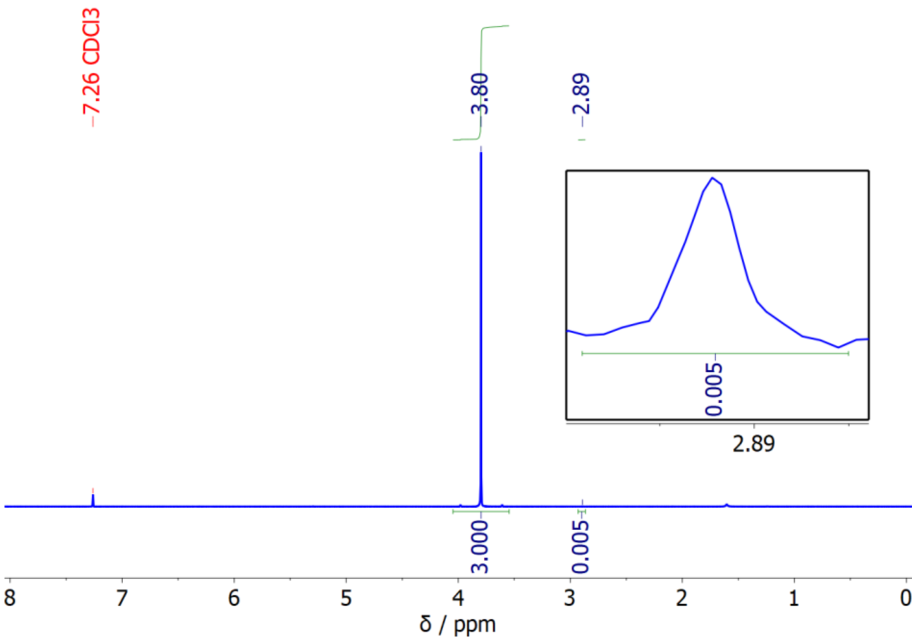

Figure S2. ^{1}H NMR Spectrum of **MP-d** after 3 exchange rounds. Alkyne proton signal of the residual protiated methyl propiolate is shown in the inset and amounts to 0.5%.

S1.4 Methods

S1.4.1 Steady-State IR Spectroscopy

Stationary IR spectra were measured in a transmission mode on Bruker Vertex 80v Fourier transform infrared (FTIR) spectrometer purged with nitrogen gas. The samples were held between two 2 mm thick CaF_2 windows in a homebuilt brass flow cell[S3] with a laser-cut PTFE spacer of typically 50 μm thickness (200 μm for some samples of the deuterated propiolate, such as in AC, CHX, CHXene, DEE, DiPrE, HFP, MeBu, NFB, TBME, THF, pXYL). Concentration of the solute was typically kept at 100 mM (500 mM for the same samples of the deuterated propiolate for which the thicker spacer was used), but other concentrations were also explored to ensure that no concentration-dependent features were present (**Figs. S6-S7**).

An average of 64 individual spectra with a spectral resolution of 0.5 cm^{-1} was obtained for each measurement. The background was taken against the empty sample compartment, and solvent spectrum was taken before each solution measurement. Solvent was removed and solution injected via a PTFE tube connected to the flow cell with the help of a syringe without opening the sample compartment or touching the cell. Direct subtraction of solvent spectrum from solution spectrum was carried out without any scaling or intensity matching. This ensures acquisition of reproducible high-quality spectra even at very low absorbances of the solute (< 1 mΔOD).

S1.4.2 NMR Spectroscopy

500 MHz Bruker AVANCE NEO NMR spectrometer equipped with a BBO cryoprobe was used for ^{1}H and ^{13}C NMR measurements. For NMR spectroscopy, samples of methyl propiolate in non-deuterated solvents (**Table S1)** were prepared typically at 100 mM concentration. A glass capillary filled with a D_2O solution of 3-(trimethylsilyl)propionic-2,2,3,3-d_4 acid sodium salt (TSP-d_4-Na) was inserted into a sample NMR

tube as a reference and deuterium lock for all ^{1}H, ^{13}C chemical shift and 2D NMR measurements. Given the large range of solvents environments probed, HSQC and HMBC ^{1}H-^{13}C spectra were acquired in any solvents whenever the ambiguity of the peak assignments was arising (e.g., due to the solvent or small admixture signals, typically in ^{1}H spectra) to ensure correct assignments of all carbon and proton peaks in 1D NMR spectra.

S1.4.3 Terahertz (THz) Spectroscopy

The time-domain THz profiles were obtained utilizing a custom-built rapid-scan multichannel THz spectrometer, described in ref. S4. In brief, the output of an amplified 100 kHz Yb-laser system (short-pulse Tangerine, Amplitude), operating at a center wavelength of 1030 nm with a pulse duration of 130 fs, is split into two branches: one for THz pulse generation and the other for THz detection (gated beam). Broadband THz pulses covering frequencies of up to 6 THz are generated through optical rectification in a 200 μm thick (110) GaP crystal. The generated THz field is focused onto the sample and then directed to the detection crystal (200 μm thick (110) GaP crystal) using two custom-made aluminum elliptical mirrors (2f = 83 mm). To facilitate multichannel detection, the gated beam is expanded 16 times and then passed through two 15×15 mm^2 BK7 glass echelon masks with variable thickness, forming a six-by-six array of beamlets that spans a total delay range of ~1.4 ps before reaching the detection crystal.[S5] Subsequently, the gated beam is imaged onto a pair of home-built photodetectors[S6] composed of 32 silicon photodiodes arranged spatially to match the shape of the beamlets. The change in polarization across all pixels of the gated beam, associated with the strength of the THz field, was measured using a small-bias electro-optic sampling detection scheme.[S7,S8] To compensate for nonuniformities between different beamlets, such as delay, noise, and intensity, the gated beam delay time was continuously varied using electromagnetically driven stage (V-408, Physik Instrumente). This method enabled the rapid acquisition of 32 time-shifted THz profiles, which were subsequently appended to generate an extended and uniform THz profile. By combining this detection scheme with the multichannel approach, so-called B-matrix referencing, as described in ref. S9, the spectrometer's signal-to-noise ratio is further enhanced.

The time-domain profiles obtained using this setup were then used to derive the THz absorption spectrum. Specifically, the spectrum was obtained utilizing two consecutive measurements of the transmitted THz fields with and without the sample. The ratio of their complex Fourier transforms was used to determine the absorption spectrum, following the procedure described in detail in Ref. S10. The resolution of these spectra is up to ~5 cm^{-1}, limited by Fabry–Perot reflections of the 200 μm-thick GaP crystals.

The sample was contained in the static cuvette made of two 200 μm sapphire windows (UQG optics) separated by a Teflon spacer with a variable thickness from 50 to 360 μm for different samples. The THz setup was enclosed within a nitrogen-purged box to prevent water vapor absorption.

S1.4.4 Molecular Dynamics Simulations

Preliminary calculations performed using ωB97x-D/def2-TZVPP[S11,S12] level of theory showed that the free-energy difference between gas-phase *trans*- and *cis*- conformers of **MP** is –18.2 kJ/mol which corresponds to 99.93% of *trans*-**MP** in the mixture of the two conformers at 293 K. Molecular dynamics (MD) simulations were performed for both *trans*- and *cis*- conformers of **MP** but the simulation results reported in the main text are for *trans*-**MP**. The structural analysis was done for both conformers and can be found in **Figs. S23-S27**. The geometry of **MP** was optimized using Gaussian 16[S13] electronic structure software package at the Hartree–Fock (HF)/6-31+G(d,p)[S14–S16] level of theory. 10 **MP** molecules were placed in a cubic box of length 30 Å and solvent molecules were added using Packmol[S17] such that the concentration of **MP** is the same as the experimental concentration for THz spectroscopy, ~10% by volume. **Table S2**

summarizes the number of molecules in the simulation box for each solvent. Subsequently, classical MD simulations were performed using GROMACS version 2024.2.[S18] Each system was energy minimized using the steepest descent algorithm.[S19] The all-atom Optimized Potentials for Liquid Simulations (OPLS-AA) force field was used.[S20] The particle mesh Ewald (PME) summation method[S21–S23] was used to describe the long-range electrostatics. A cutoff of 1 nm was applied for both electrostatic and van der Waals interactions. The equations of motion were integrated using the Verlet algorithm.[S24] The energy minimized configurations were equilibrated in two stages. First, a 100 ps NVT equilibration was performed at 293 K using the velocity-rescale (v-rescale) thermostat. Second, a 100 ps NPT equilibration at 1 bar using the Parrinello–Rahman barostat was performed. Further, the classical MD trajectories were used to extract equilibrated configurations which served as initial structures for subsequent *ab initio* molecular dynamics (AIMD) simulations. Quickstep[S25] module of the CP2K software package was used for AIMD simulations.[S26] Becke–Lee–Yang–Parr (BLYP) exchange-correlation functional[S27,S28] with Grimme's D3 empirical dispersion[S29] correction were used in AIMD simulations. The core electrons of all atoms were treated using the dual-space norm-conserving Goedecker–Teter–Hutter (GTH) pseudopotentials.[S30,S31] A combined Gaussian and plane wave (GPW) framework[S25,32] was used with a triple-ζ valence doubly polarized (TZV2P) basis set[S12,S33,S34] and a plane wave cutoff of 400 Ry. The systems were first equilibrated under isothermal–isochoric (NVT) conditions for 10 ps. Temperature was maintained at 293 K using the Nosé–Hoover thermostat.[S35,S36] The equilibration step was followed by a 50 ps production simulation under microcanonical (NVE) conditions. The time-step for the equation of motion was set to 0.5 fs. Three-dimensional periodic boundary conditions were employed. Trajectories were saved every 0.5 fs and were used to compute the terahertz (THz) spectra.

The vibrational density of states (VDOS) reported in **Fig. 6b** in the main text was obtained by the Fourier transform of the normalized velocity autocorrelation function (VACF) $C_{vv}(t) = \langle v(0)\, v(t)\rangle / \langle v(0)^2\rangle$, where $v(t) = \dot{r}_{\mathrm{H}}(t)$ is the instantaneous velocity of the alkynyl H atom of *trans*-**MP** calculated from AIMD trajectories.

To quantify the local electrostatic environment of the C–H group of **MP**, we calculated the electric field at the H atom of the alkynyl carbon and projected it onto the corresponding C–H bond: $E = \hat{r}_{CH} \cdot \sum_i \frac{q_i}{r_{iH}^2} \hat{r}_{iH}$, where $\hat{r}_{CH}$ is the unit vector that points from alkynyl C to H atoms of **MP**, r_{iH} is the distance (in a.u.) between the ith atom of a solvent molecule and the alkynyl H atom of **MP** ($\hat{r}_{iH}$ is the corresponding unit vector), q_i is the charge (in a.u.) of ith solvent atom, and the sum goes over all charged atoms of the solvent molecules within 20 Å of the alkynyl hydrogen atom of **MP**. The atomic charges were calculated from a 50 ps AIMD trajectory. Two types of atomic partial charges were examined: electrostatic potential (ESP)[S37,38] derived and Mulliken charges.[S39]

Table S2. The composition of simulation boxes for each system.

Number of *trans*-MP molecules	Solvent	Number of solvent molecules	Total number of molecules
10	Acetone	121	131
	DBE	52	62
	THF	120	130
	DMSO	125	135
	ACN	170	180
	Cyclohexane	82	92

S1.4.5 Quantum Chemical Calculations

Several geometry optimizations and vibrational frequency calculations were performed on isolated molecules/H-bonded complexes at DFT level of theory. Calculations were performed with Gaussian 16 package[S13] using ωB97X-D[S11] functional and 6-311++G(d,p) basis set. Solvation was included using polarizable continuum model.[S40] The minimum of the structure optimization was validated by the absence of imaginary frequencies in the computed vibrational normal modes. The vibrational frequencies were scaled by an empirical factor of 0.98 determined to best fit the experimental vibrational frequencies of **MP**.

Total interaction energy and its physical components for the two complexes of **MP** with EtAc and THF were calculated using Symmetry Adapted Perturbation Theory (SAPT).[S41] First, the geometry of each complex was optimized at MP2/aug-cc-pVDZ level using resolution of identity integral approximation.[S42–S44] Then SAPT calculation was performed for the optimized complex geometry using SAPT2+(3)δMP2/aug-cc-pVTZ level of theory. The results are summarized in **Table S8**. SAPT2+(3)δMP2 is referred to as the gold standard in SAPT applications.[S45] All calculations were performed using Psi4 software package version 1.9.1.[S46]

S2 ADDITIONAL SPECTROSCOPIC DATA AND DETAILS

S2.1 Calculated Structures and Frequencies of H-bonded MP

The structure of **MP**, as well as several of its exemplary hydrogen-bonded complexes with several H-bond accepting solvents have been optimized in vacuum to test whether the experimentally observed vibrational redshift is reproduced by quantum-chemical calculations. These structures are shown in **Fig. S3**, and **Table S3** lists obtained ≡C–H stretch vibrational frequencies compared to the experimental values. While these snapshots are not necessarily representative of real liquid solution structures and serve rather as limiting illustrative examples, they demonstrate that the experimentally observed redshift and its magnitude is well-reproduced with this simple approach.

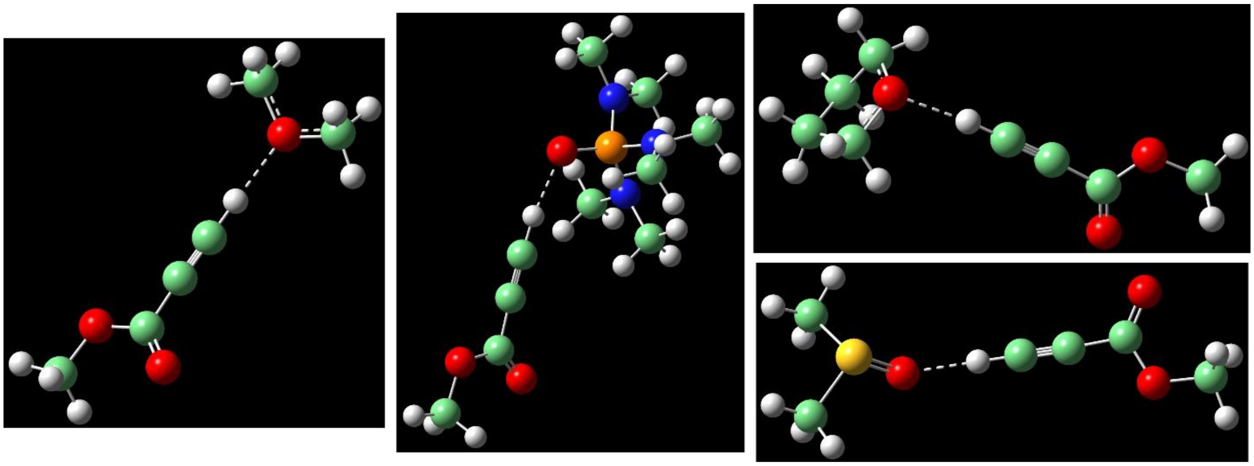

Figure S3. Optimized structures of **MP** with several exemplary H-bond accepting solvents: ether (left, taken as the simplest representative of the class – dimethyl ether), HMPA (middle), THF (right, top), DMSO (right, bottom). C–H hydrogen bond to a heteroatom is shown as a dashed line. The atoms are colored as follows: green (carbon), red (oxygen), blue (nitrogen), yellow (sulfur), orange (phosphorus), grey (hydrogen).

Table S3. Comparison of experimentally observed versus quantum chemically calculated frequencies for H-bonded complexes shown in **Fig. S3**.

H-bond Acceptor	Calculated Frequency / cm^{-1}	Experimental Frequency / cm^{-1}
none	3412*	3384**
non-cyclic ether	3222	3218
THF	3191	3202
DMSO	3146	3148
HMPA	3139	3086

* Calculated in vacuum.

** This is an estimated frequency in gas-phase obtained from the quadratic fit of the experimental dependence of the non-H-bonded C–H frequency on dispersion in **Fig. 3b**.

S2.2 Fermi Resonance in Protiated MP

We have not investigated in detail the exact nature of the Fermi resonance band borrowing intensity from the free C–H, but we speculate that this band is likely the combination band of the C–H bending vibration first overtone and the C≡C stretch. Our simple quantum-chemical anharmonic calculations indicate that this combination band is at lower frequencies in both protiated and deuterated propiolate. This mode assignment is in line with previous spectroscopic work.[S47] Experimentally, it also persists both in other terminal alkynes[S47,S48] and in a deuterated variant. We opted for a solvent perturbation of Fermi resonance to test whether the solvent variation of the free C–H can explain the increased spectral intensity of the π band in CHXene.

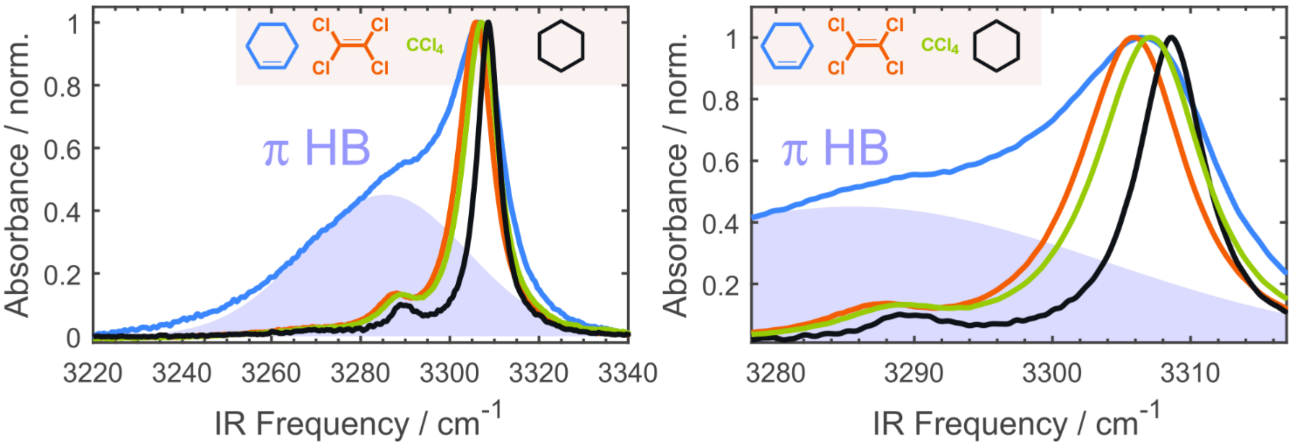

Figure S4. C–H IR spectra of **MP** in carbon tetrachloride, cyclohexene and tetrachloroethylene compared to the spectrum in cyclohexane. Extracted π HB band in CHXene is shown as a shaded blue band. The free C–H in CCl_4 and TCE occur at essentially identical peak positions as in CHXene and their respective Fermi resonance band is only weakly perturbed in both solvents compared to CHX.

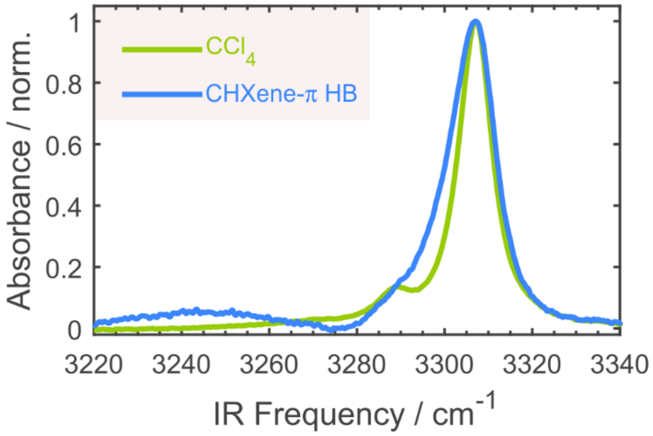

Figure S5. C–H IR spectra of **MP** in carbon tetrachloride compared to the spectrum in CHXene with its π HB band subtracted. The free C–H occurs at essentially identical peak position and a shoulder of the weak Fermi resonance band is also observed at the same position in both solvents.

Table S4. Ratio of integral intensities of the Fermi resonance band (FR) to the free C–H stretch for a few selected solvents where the position of the free C–H is essentially identical as in CHXene. The spectrum was decomposed using two Gaussian bands and their respective integrals were taken to quantify integral intensities. CHX is shown as a reference.

Solvent	Intensity ratio (FR/C–H)	FR peak / cm^{-1}	Free C–H peak / cm^{-1}
CHX	0.07	3289.5	3308.6
CCl_4	0.07	3286.6	3305.8
TCE	0.06	3287.8	3307.0
CHXene	1.53	3285.5	3306.2

S2.3 Concentration Dependence and Aggregation Effects

Aggregation of **MP** occurs upon increase of its concentration above 100 mM only in non-polar and very weakly polar solvents (e.g., hydrocarbons, di-*n*-butyl ether). In medium and highly polar solvents solute-solvent interactions outcompete solute-solute interactions between propiolate molecules and no signs of aggregation are observed in the FTIR spectra up to at least 500 mM concentration (higher concentrations were not monitored).

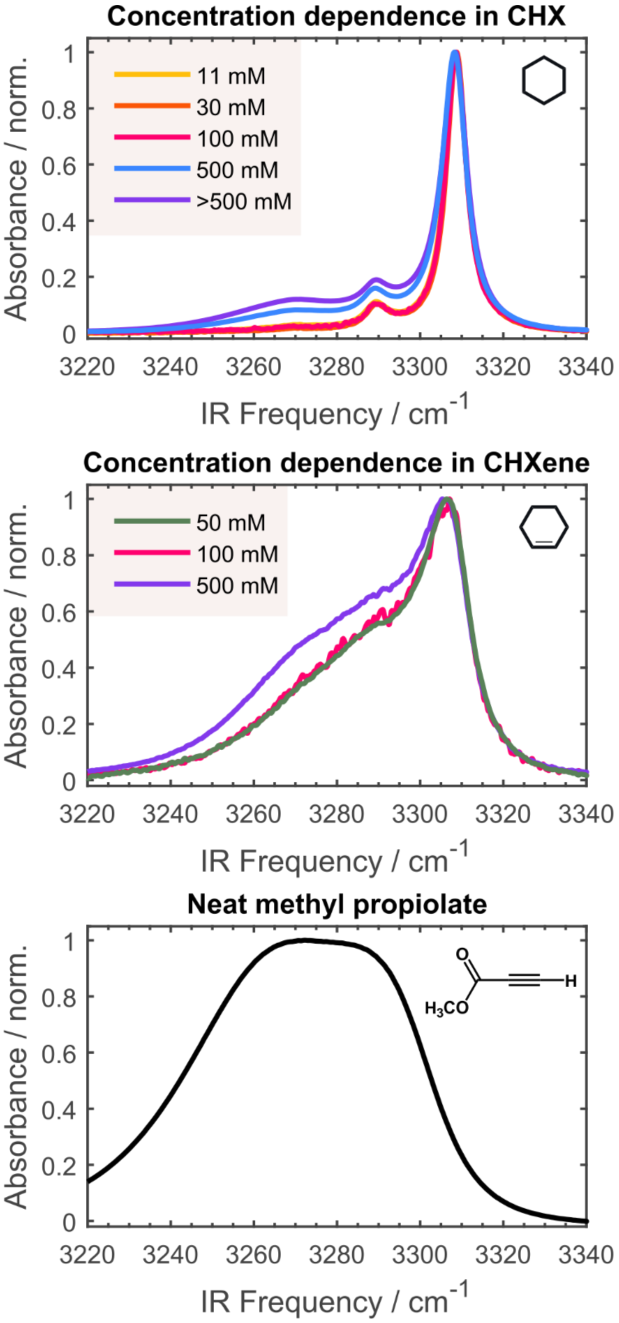

Figure S6. C–H IR spectra of **MP** recorded at different concentrations in cyclohexane (top), cyclohexene (middle) and of neat liquid (bottom). The spectrum of neat liquid was recorded without any spacer between two CaF_2 windows, and the maximum absorption in this region did not exceed OD = 0.5.

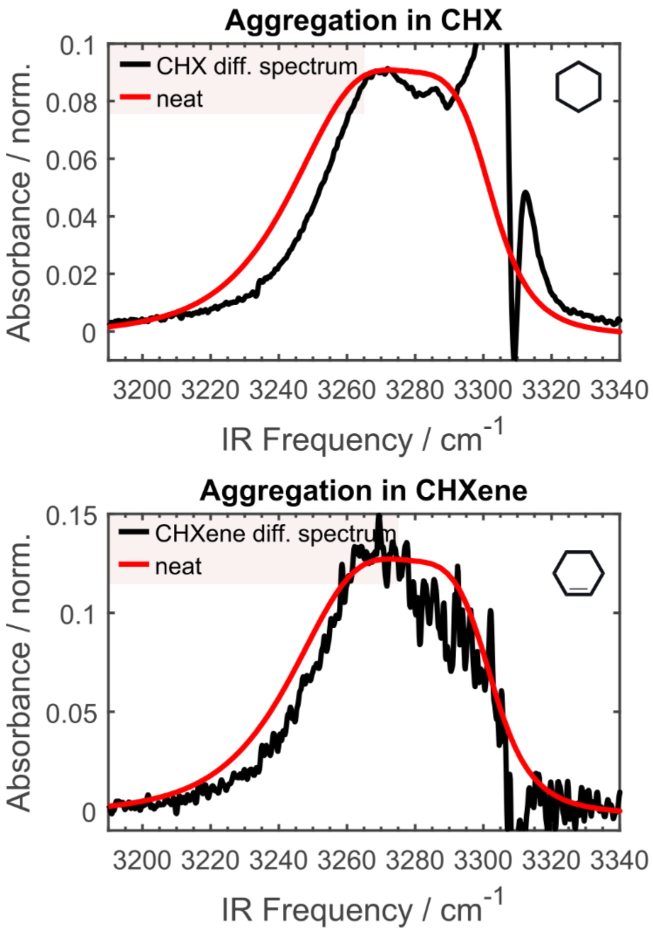

Figure S7. C–H IR difference spectra of high concentration (500 mM) and dilute (30-50 mM) spectra of **MP** in cyclohexane (top) and cyclohexene (bottom) shown as black lines compared to the scaled spectrum of neat methyl propiolate (**Fig. S6**) shown as a red line.

S2.4 π-π Interactions

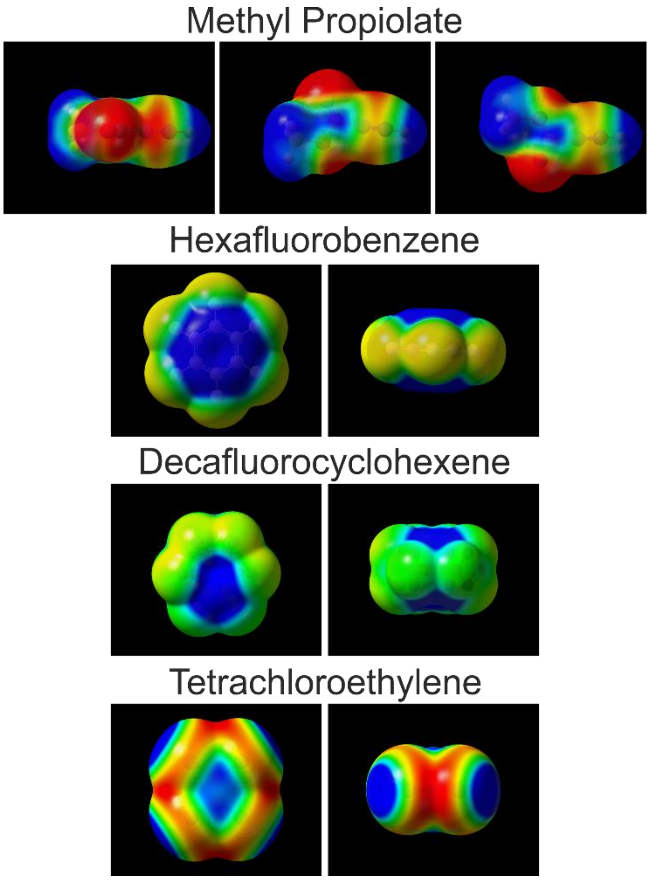

Figure S8. Molecular electrostatic surface potential of **MP** along with a few electron-depleted π-systems shown from different sides. Blue color represents positive regions, while red color corresponds to negative regions.

Molecular electrostatic surface potential clearly shows a ring of negative region around the triple bond, while perfluorinated benzene and cyclohexene along with perchlorinated ethylene (but to a lesser extent) feature strong π-acidic surfaces above and below the corresponding unsaturated moieties. If π-π stacking interactions were operative between **MP** and unsaturated solvents, these electrostatic effects would strongly favor them. Nevertheless, the experiments demonstrate that no specific interactions occur in these systems.

Also, while we located a hydrogen-bonded complex geometry between **MP** and cyclohexene (**Fig. S9**), our attempts to optimize an alternative π-π stack geometry when starting from various pre-aligned arrangements of the π-systems of **MP** and cyclohexene, failed even in vacuum.

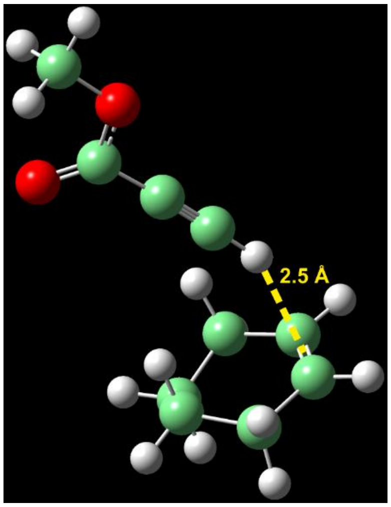

Figure S9. Quantum chemically optimized hydrogen-bonded structure of the **MP**/CHXene complex in vacuum. The C–H…single π hydrogen bond with its tentative length is shown in yellow. We warn against taking this value and geometry literally, as the calculations in vacuum may at best represent only a limiting case, or one possible conformation, of the distribution of structures that exist in the liquid phase. The atoms are colored as follows: green (carbon), red (oxygen), grey (hydrogen).

Table S5. C≡C Stretch vibrational frequency of **MP** and its derivatives substituted on the alkyne carbon with methyl and trimethylsilyl groups in cyclohexane, cyclohexene and both conjugated 1,3-and non-conjugated 1,4-cyclohexadienes. The absolute values, as well as frequency differences between the respective solvent and cyclohexane, are indicated for each molecule. The frequencies were obtained from the Gaussian fit near the maximum of the band.

**Solvent**	**–C≡C–H / cm^{-1}**		**–C≡C–CH_3 / cm^{-1}**		**–C≡C–$Si(CH_3)_3$ / cm^{-1}**	
CHX	2129.43	reference	2251.58	reference	2179.10	reference
CHXene	2128.15	-1.28	2250.30	-1.28	2178.46	-0.64
CHX2ene-1,3	2127.18	-2.25	2249.01	-2.57	2177.45	-1.65
CHX2ene-1,4	2126.83	-2.60	2248.72	-2.86	2177.30	-1.80

Table S6. C=O Stretch vibrational frequency of **MP** and its derivatives substituted on the alkyne carbon with methyl and trimethylsilyl groups in cyclohexane, cyclohexene and both conjugated 1,3-and non-conjugated 1,4-cyclohexadienes. The absolute values, as well as frequency differences between the respective solvent and cyclohexane, are indicated for each molecule. The frequencies were obtained from the Gaussian fit near the maximum of the band.

**Solvent**	**C=O (in ≡C–H) / cm^{-1}**		**C=O (in ≡C–CH_3)/ cm^{-1}**		**C=O (in ≡C–$Si(CH_3)_3$) / cm^{-1}**	
CHX	1731.80	reference	1727.62	reference	1723.76	reference
CHXene	1731.36	-0.44	1723.12	-4.50	1722.38	-1.38
CHX2ene-1,3	1728.11	-3.69	1719.27*	-8.35	1719.95	-3.81
CHX2ene-1,4	1727.75	-4.05	1719.16**	-8.46	1719.71	-4.05

* The indicated maximum is an apparent maximum. This band consists of a band at 1718.85 cm^{-1} and a shoulder at 1729.11 cm^{-1} if decomposed into two Gaussian lineshapes.

** The indicated maximum is an apparent maximum. This band consists of a band at 1718.04 cm^{-1} and a shoulder at 1721.76 cm^{-1} if decomposed into two Gaussian lineshapes.

From **Tables S5-S6** we can see that the effect of changing solvent from CHX to CHXene or CHX2ene affects vibrational frequencies of the alkyne and carbonyl stretch only very slightly (by about 1-2 cm^{-1}). The magnitude of this shift is much smaller than for C-H stretch and it does not meaningfully decrease upon increasing the bulkiness of the substituent on the alkynyl carbon. As such, it does not originate from the π-π stacking type of interactions between alkynes and alkenes. Instead, this small effect is consistent with the weak non-specific electric field/dispersion effect as described in the main text.

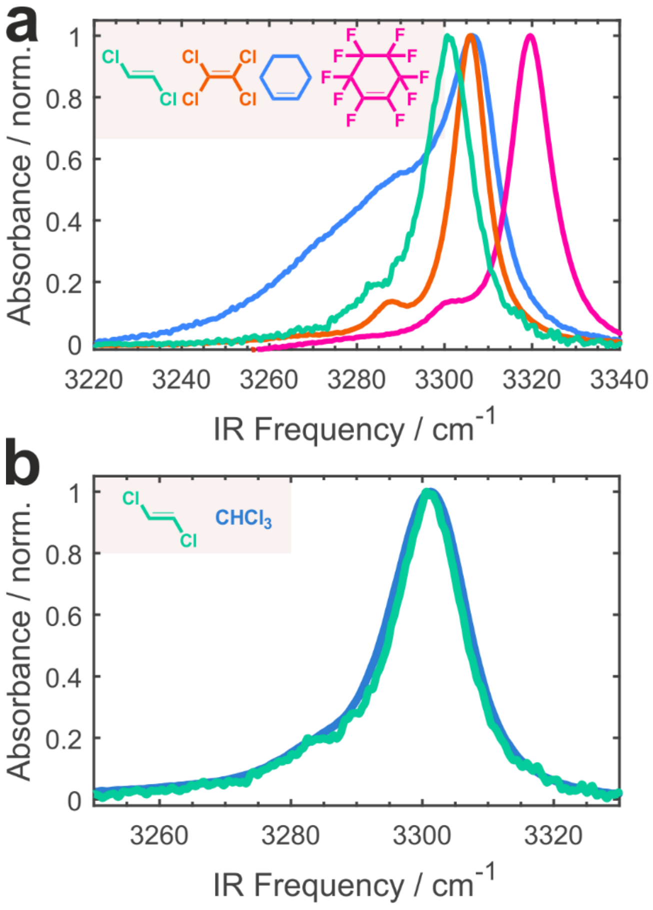

Figure S10. Searching the limit of C–H-single π H-bonding: **a**) ≡C–H IR spectra of **MP** in solvents with single carbon-carbon double bonds: cyclohexene (CHXene), perfluorinated cyclohexene (CHXene-F_{10}), perchlorinated ethylene ($Cl_2C{=}CCl_2$), *trans*-dichloroethylene (ClHC=CHCl, DCE). **b**) ≡C–H IR spectra in chloroform and *trans*-dichloroethylene (DCE) are identical. The free C–H band is accompanied by the weak Fermi resonance band. No specific interactions between **MP** and electron density depleted double bond in DCE are observable.

S2.5 Free and H-bonded C–H

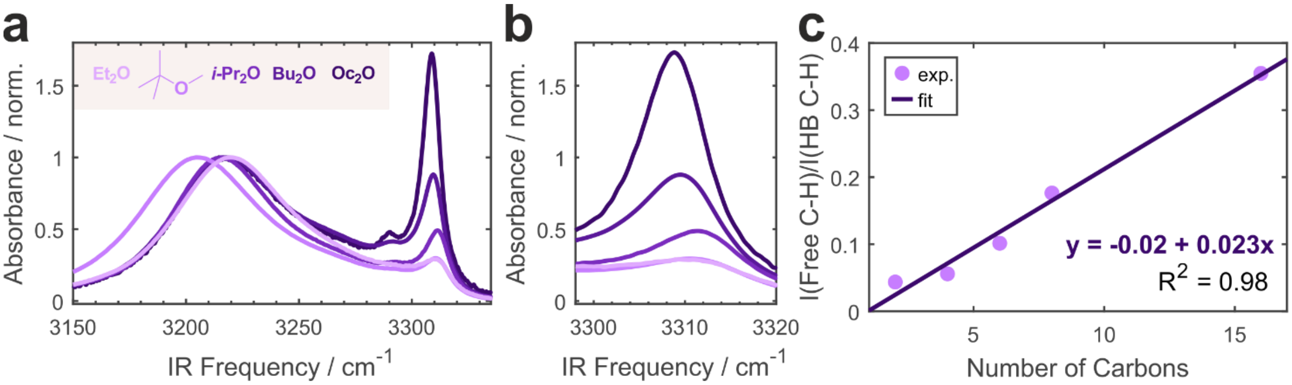

Figure S11. a), **b)** C–H IR spectra of **MP** in various non-cyclic ethers. Besides the band of the free C–H at 3300-3315 cm^{-1} (zoomed into in panel **b**) and its accompanying Fermi resonance at 3290 cm^{-1}, the broad red-shifted band of the H-bonded C–H at 3150-3250 cm^{-1} dominates spectra. Spectra are normalized to the maximum of this band. **c)** The growth of the free C–H band intensity is linear with the total number of carbons in ether alkyl chains due to the entropic factor of HB formation. Experimental data points are shown as markers. They were extracted by decomposing the total spectrum into its constituent bands (see **Fig. S14** and main text for explanation behind the number of bands and their nature), and taking the ratio of integral intensities of the free C–H band to the hydrogen-bonded C–H. Linear fit is shown as a line along with its estimated coefficients and R^2.

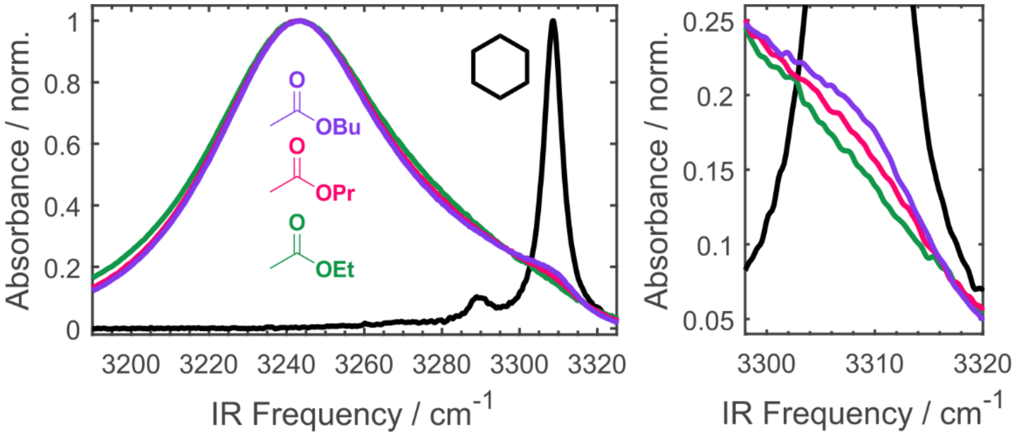

Figure S12. Normalized C–H IR spectra of **MP** in several esters with growing side chain length: ethyl acetate (2 carbons), propyl acetate (3 carbons), butyl acetate (4 carbons) compared to cyclohexane. The H-bonded peak remains identical as the hydrogen bond accepting ability of these esters is preserved, but the growing length of the alkyl tail causes entropic penalty for HB formation, and the free C–H consistently grows by the same amount as the chain length is augmented by each additional methylene unit. This trend for esters is similar to and consistent with the one for ethers shown in **Fig. S11**.

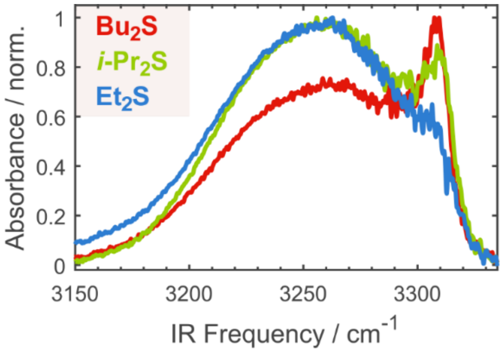

Figure S13. Normalized C–H IR spectra of **MP** in several sulfides with varying side chain length: diethyl sulfide (2 carbons), di-isopropyl sulfide (3 carbons), di-*n*-butyl sulfide (4 carbons). The H-bonded peak remains identical as the hydrogen bond accepting ability of these thioethers is preserved, but the growing length of the alkyl tail causes entropic penalty for HB formation, and the free C–H consistently grows similar to the trend shown for ethers in **Fig. S11**.

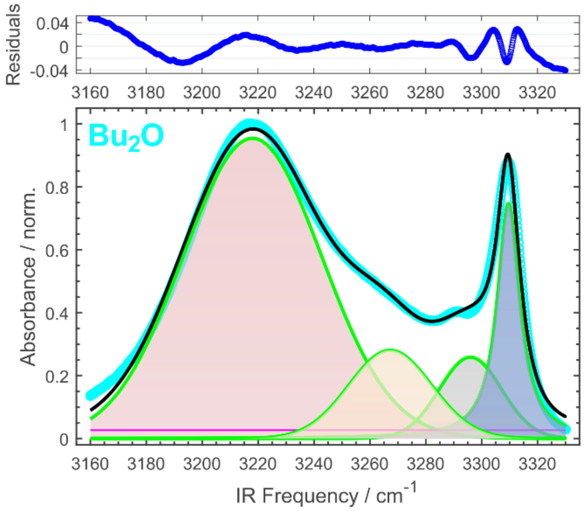

Figure S14. C–H IR spectrum of **MP** in di-*n*-butyl ether decomposed into individual bands whose peak positions can be found in **Table S6** (highlighted in green). The experimental spectrum is shown with cyan markers, green lines show outlines of the individual bands and each band is shaded with its own color (see **Fig. 1c** or **S15c** for the color code); the background is represented with a magenta line; the total fit is shown as a black line. Fit residuals are blue markers at the top.

Table S7. Decomposition of the C–H stretch vibrational spectrum of **MP** in acyclic ethers where multiple distinct populations are observed. The frequencies were obtained by fitting the spectra to a sum of the Lorentzian bandshape for the free C–H and Gaussian bands for other species. Typical fit quality can be seen in **Fig. S14** for di-*n*-butyl ether shown as an illustrative example (highlighted here in green). Bands are color-coded according to **Fig. S14**.

Solvent	Free C-H / cm^{-1}	FR / cm^{-1}	Frustrated C-H / cm^{-1}	HB C-H / cm^{-1}
Di-n-octyl ether	3308.7	3290.7	3277.6	3217.9
Di-n-butyl ether	3309.6	3296.1	3267.2	3217.9
Di-isopropyl ether	3311.6	3295.1	3265.0	3216.1
Methyl-tert-butyl ether	3311.3	3294.9	3262.5	3205.6
Diethyl ether	3311.4	3293.8	3267.3	3220.4

Fig. S15 shows temperature dependence of the C–H IR spectrum of **MP** in di-*n*-butyl ether. As temperature increases, the H-bonded C–H blueshifts due to the weakening of the intact hydrogen bonds. This leads to the characteristic sigmoidal shape in the difference spectra (ΔA = high-temperature spectrum – low-temperature spectrum) due to the loss of the spectral intensity on the red-side of the band and gain on the blue side (**Fig. S15c**). The relative intensity of the non-H-bonded C–H peak grows strongly signifying the breaking of H-bonds (**Fig. S15a, c**). Importantly, the contribution of the frustrated band also increases notably providing evidence of H-bonds breaking of C–H groups that remain near the oxygen atom and are stabilized by non-specific dipolar interactions with the solvent. This is clearly seen both in the raw data (**Fig. S15a**) and in the centered spectra (**Fig. S15b**), where the strong growth on the high-frequency wing of the H-bonded band is observed – the location of the frustrated band, as well as in the difference spectra (**Fig. S15c**).

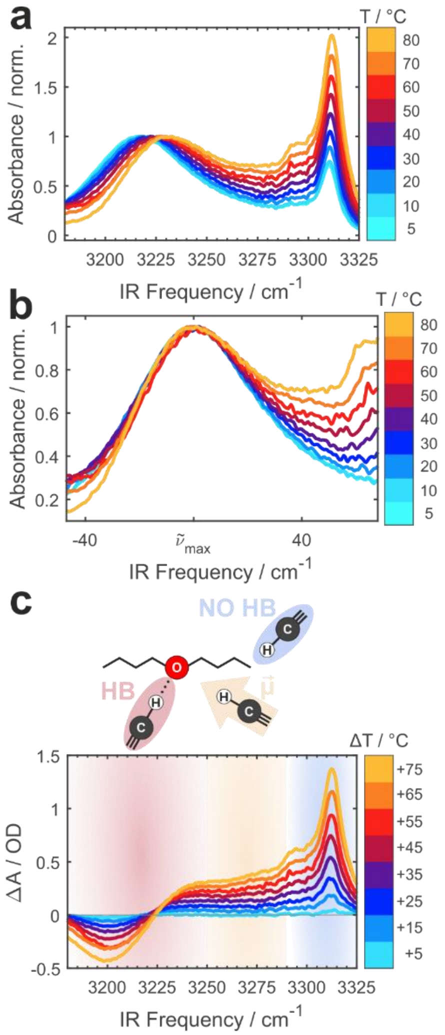

Figure S15. Temperature dependence of the C–H IR spectrum of **MP** in di-*n*-butyl ether: **a)** experimental C–H spectra recorded at different temperatures indicated in the colorbar and normalized to the maximum of the hydrogen-bonded band; **b)** same as in **a)** but centered on the hydrogen-bonded band maximum; **c)** temperature difference spectra calculated as a difference between an experimental spectrum at a given temperature and the lowest temperature (5 °C) spectrum. Temperature difference is labelled in the colorbar. Spectral changes arising from three distinct C–H species (H-bonded, frustrated, non-H-bonded) are color-coded. Scheme on top depicts the nature of these C–H species.

S2.6 C–D Stretch: Fermi Resonance and Single π Hydrogen Bonding

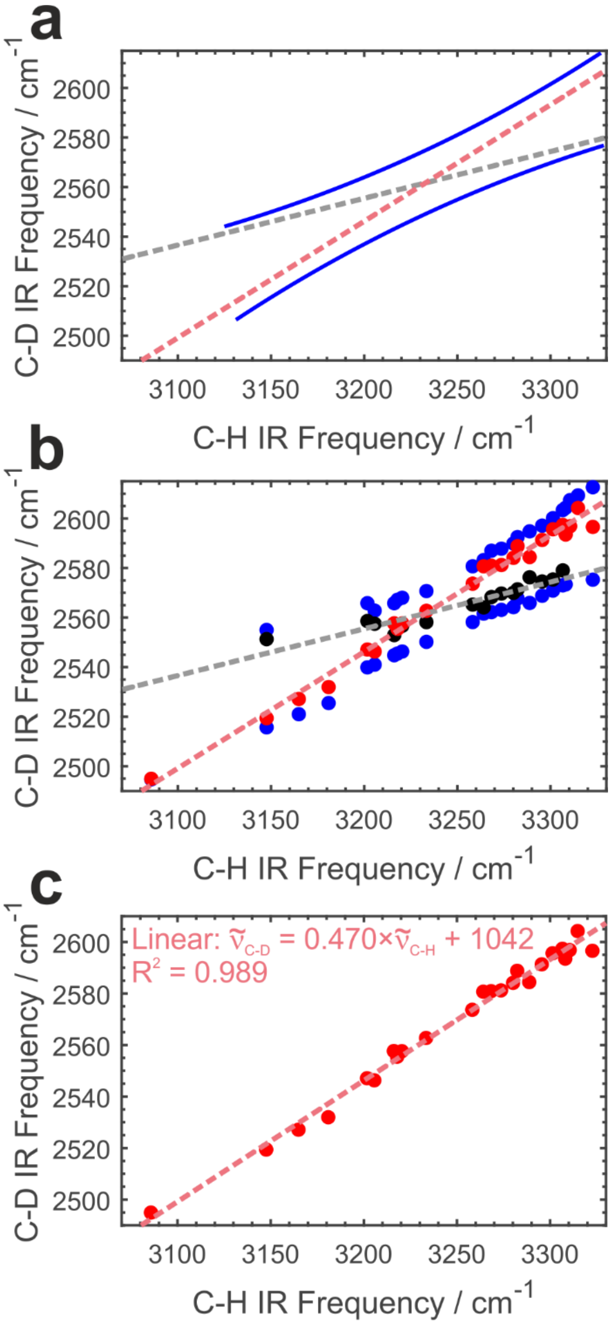

Figure S16. Deperturbing accidental Fermi resonance of the C–D stretch: **a)** schematically shown adiabatic (blue curves) and diabatic (red and grey dashed lines) states as a function of the C–H stretch frequency of the protiated molecule in the same solvents (chosen as a tuning solvent- dependent parameter since it does not engage in the accidental FR) demonstrate typical avoided-crossing behavior; **b)** experimental data shown as markers for the measured positions of the two observed bands (blue), as wells as the deperturbed C–D stretch (red) and the dark mode (black) along with their corresponding linear fits; **c)** solvent dependence of the deperturbed C–D stretch shown with its linear fit, fit equation and coefficient of determination.

C–D stretch vibration obtained upon spontaneous exchange of the alkynyl proton enters several accidental Fermi resonance interactions. In addition to the persistent FR analogous to the one observed in the protiated molecule (Section S2.2) there is another emerging FR seen in the spectral data. The most striking observation is made upon inspection of Fig. 1a, right panel, in the main text, where the H-bonded C–D stretch (e.g., in di-*n*-butyl ether) is clearly split into two bands of almost equal intensity signifying strong coupling to another vibration. The band splitting and intensity of the emerging second band decreases on both high- and low-frequency sides compared to the di-*n*-butyl ether case such that, for example, in the strongest H-bonding solvents (e.g., in hexamethylphosphoramide and DMSO), there remains a single unperturbed band.

Given this behavior reminiscent of avoided crossing (**Fig. S16a**, **b**) we used a solvent perturbation method to identify that this splitting comes from the accidental Fermi resonance with a dark combination/overtone band in this region, whose exact nature is of little relevance. We deperturbed this FR using measured frequency positions and integral intensities of both bands, which were obtained by iteratively fitting the whole C–D stretch spectral region to a sum of Gaussian and Lorentzian (for non-H-bonded band only) lineshape functions. A standard assumption that the dark state gains all its intensity exclusively from the FR with the bright C–D stretch was made. Corresponding diabats for the bright (red markers) and dark (black markers) states are shown in **Fig. S16b**. The solvent dependence of these deperturbed diabatic states was further fitted to linear functions (**Fig. S16b**, **c**). As expected, the bright C–D stretch demonstrates quantitative linear correlation with the C–H stretch ($R^2 = 0.99$) of the corresponding protiated case.

Due to the occurrence of these multiple FR interactions observed even in non-polar and non-interacting cyclohexane (**Fig. S17a**, black spectrum), observing the C–D single π H-bond to cyclohexene is less straightforward than for the protiated molecule (**Fig. 2**). **Fig. S17** shows that upon going from cyclohexane to cyclohexene the non-H-bonded C–D stretch redshifts and a new shoulder emerges on its low-frequency side peaking around 2590 cm^{-1}. Additionally, a more intense FR peak grows around 2565 cm^{-1}. In both cyclohexadienes (conjugated 1,3 and non-conjugated 1,4 variants) the spectra are very similar and the main difference with cyclohexene is the strongly reduced intensity of the free, non-H-bonded C–D stretch band while the shoulder around 2590 cm^{-1} becomes the dominant band (**Fig. S17**). Qualitatively, these spectral changes are reminiscent of the changes in the protiated molecule shown in **Fig. 2c** of the main text, but complicated by the FR interferences.

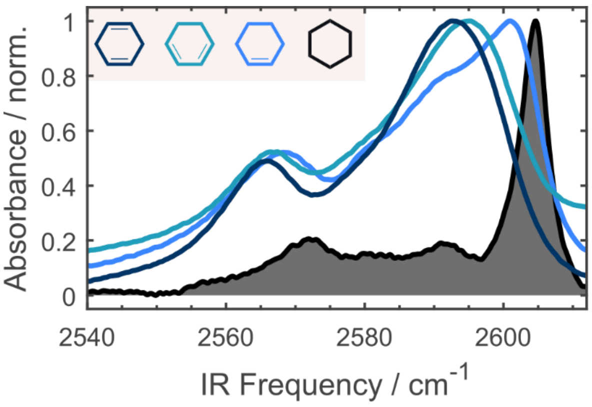

Figure S17. Infrared absorption spectra of **MP-d** in cyclohexane (black curve, shaded) and in non-polar double-bond containing solvents: cyclohexene and cyclohexadienes.

To demonstrate this unambiguously, we show in **Fig. S18a** the spectrum of **MP-d** in chloroform – the medium where no hydrogen bonding between **MP** and solvent takes place and where the main C–D band almost exactly coincides with the non-H-bonded peak observed in cyclohexene. To compensate for a minute difference in peak positions we further shift the chloroform spectrum by 0.3 cm^{-1} to the blue. Then we subtract this intensity-matched spectrum from the one in cyclohexene to separate the contribution of the hydrogen-bonded **MP-d** to a single carbon-carbon double bond. This contribution is highlighted as a pink-shaded area in both panels **a** and **b** in **Fig. S18**. We further compare this contribution with **MP-d** spectrum in cyclohexadiene-1,4 scaled by a factor of 0.5 to account for the entropic difference (due to the halved accessible π surface area) in the free energy of H-bond formation as noted in the main text for **MP**. As one can see, the correspondence between the two spectra in **Fig. S18b** is very good. We note that the major difference between the two spectra comes from the fact that cyclohexadiene still contains a notable amount of non-H-bonded **MP-d**, whose contribution is significantly reduced compared to cyclohexene but is not negligible. Since we do not decompose this contribution for the same reason as we did not do it for protiated **MP**, this likely accounts for the difference on the blue side of the main band and its corresponding Fermi resonance peak around 2575 cm^{-1}. Overall, these additional data provide further evidence of unconventional and unexpected single π H-bonding in between C–D and C=C bonds.

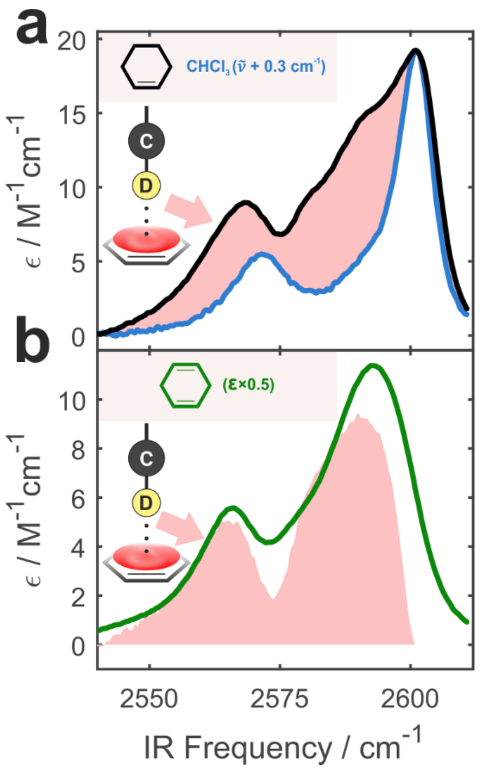

Figure S18. Infrared molar absorption coefficient spectra in the C–D stretch spectral region of **MP-d**: **a)** in cyclohexene (black line) and in chloroform (blue line, additionally shifted to high frequencies by 0.3 cm^{-1}) – the difference between the two spectra corresponds to the contribution of the single π H-bonded **MP-d** shown in **b)** as a pink shaded spectrum along with the spectrum in cyclohexadiene-1,4 scaled by factor of 0.5 (green line) to account for twice as low π-surface area of cyclohexene.

S2.7 Non-Electrostatic Contribution vs. Gutmann Donor Number

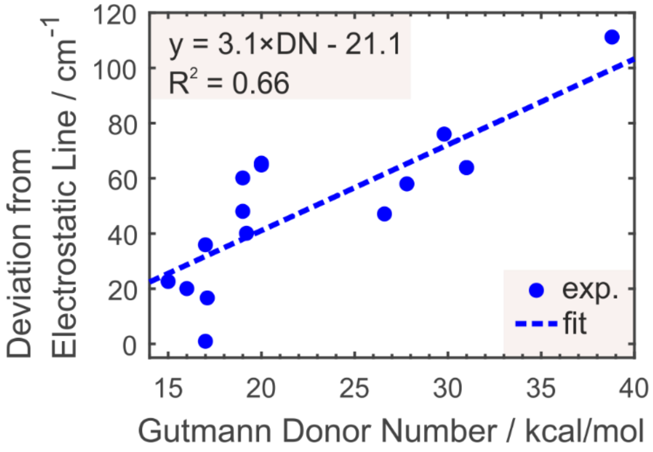

Figure S19. Correlation between the deviation of the ≡C–H stretch frequency from the electrostatic line depicted in **Fig. 5b** and Gutmann donor number (DN) for each of the strongly H-bond accepting solvents. The linear fit is shown along with its equation and coefficient of determination. We note that the fit coefficient for DN is essentially identical to that reported by Boxer, Markland and co-workers (3.4 in their case),[S48] where it was produced based on computational data.

S2.8 THz Interaction Spectra

Density-induced solution non-idealities

THz interaction spectra presented in **Fig. 6a** in the main text were calculated as

$$\alpha^{int} = \alpha^{exp} - \alpha^{ideal},$$

where α^{exp} is the experimentally determined quantity and

$$\alpha^{ideal} = x_{solute}\alpha_{solute} + x_{solvent}\alpha_{solvent}$$

with α representing the pathlength-corrected absorbance, which is a standard metric in THz absorption spectroscopy. Quantities α_{solute} and $\alpha_{solvent}$ were measured experimentally for neat liquids. Unlike for UV-vis and IR absorption spectroscopy, where ε, the molar extinction coefficient, is usually a common metric of interest, α nominally represents a product of ε and molar concentration c. This is because c is often ill-defined in the THz regime due to the highly delocalized nature of the intermolecular modes spanning many solvent and solute molecules. As such, density-dependent changes are accounted for in the interaction spectrum inasmuch as solution behaves as an ideal mixture. Solution non-idealities, such as excess molar volume (V_m^{exc}), would still show up in the interaction spectrum since α is proportional to concentration and thus inversely proportional to molar volume V_m

$$\alpha^{int} \propto \frac{1}{V_m}$$

$$V_m = V_m^{ideal} + V_m^{exc}$$

$$\frac{1}{V_m} = \frac{1}{V_m^{ideal} + V_m^{exc}} = \frac{1}{V_m^{ideal}}\left(\frac{1}{1 + \frac{V_m^{exc}}{V_m^{ideal}}}\right)$$

Therefore, density-induced solution non-idealities affect α^{int} via a small parameter $\frac{V_m^{exc}}{V_m^{ideal}}$. The factor in parentheses can be approximated to the first order as

$$\frac{1}{1 + \frac{V_m^{exc}}{V_m^{ideal}}} \approx 1 - \frac{V_m^{exc}}{V_m^{ideal}}$$

leading to

$$\frac{1}{V_m} \approx \frac{1}{V_m^{ideal}}\left(1 - \frac{V_m^{exc}}{V_m^{ideal}}\right)$$

$$\alpha^{exp} \approx \alpha^{ideal}\left(1 - \frac{V_m^{exc}}{V_m^{ideal}}\right)$$

$$\alpha_{dens}^{int} = \alpha^{exp} - \alpha^{ideal} \approx -\frac{V_m^{exc}}{V_m^{ideal}}\alpha^{ideal}$$

with α_{dens}^{int} signifying the density-induced contribution to the THz interaction spectrum arising from non-idealities. We have measured the densities of both neat **MP** and neat solvents, as well as **MP** solutions and calculated the molar volumes and excess molar volumes. For example, in di-*n*-butyl ether and acetone, the two examples, where the magnitudes of α^{ideal} are among the largest reaching up to 200-400 cm^{-1} at the spectral maximum within our probing window, $\frac{V_m^{exc}}{V_m^{ideal}} = 0.004$ and 0.0167 respectively. Such magnitude of density non-ideality is common. Thus, the density-induced deviations could be conservatively estimated $\alpha_{dens}^{int} = 1 - 5$ cm^{-1}. This magnitude is almost two orders of magnitude smaller than the observed values of α^{int} in these solvents. Moreover, what is more important is that the excess molar volume is positive meaning that these deviations decrease the interaction spectrum features instead of potentially spuriously increasing the new contributions. Therefore, we conclude that density-induced deviations are both qualitatively and quantitatively inconsistent with the observed spectral changes.

Control experiments in non-H-bonding solvents

Three control experiments were carried out using solvents that do not form the hydrogen bond in question. Both cyclohexane and isooctane hydrocarbons as non-polar solvents incapable of any hydrogen bonding and featuring different densities and viscosities were used. In addition, chloroform, as a medium polar solvent that can only act as an H-bond donor but not acceptor was used as well. All three controls demonstrated qualitatively different behavior than other 7 media, where C(sp)–H hydrogen bonding occurs. While in hydrocarbons the response is negligibly small, in chloroform the interaction spectrum comprises a negative central feature flanked by two positive bands – all having the same intensity, and thus signifies broadening of the solvent phonon band.

We emphasize that all the data in **Fig. 6a** are presented on the same absolute scale (indicated by the vertical bar in the top left corner of the panel) and are only offset vertically by the same amount for clarity. Clearly,

not only do all three controls exhibit qualitatively different behavior, but also the magnitude of their response is significantly smaller than in H-bond forming media.

Frequency shifts and AIMD simulations

Both emerging bands in H-bond accepting solvents follow the trends expected for the emerging hydrogen-bond stretching and bending modes: the bend mode redshifts to lower frequencies as the hydrogen bond strengthens, while the stretch mode shifts to the blue. This behavior is opposite to the one observed for the intramolecular stretch and bend modes (redshifting stretch and blueshifting bend), as is known from numerous studies on O–H spectroscopy as a common proper H-bond donor.

These spectral trends are further confirmed by AIMD simulations (**Fig. 6b**), where the vibrational density of states (VDOS) in the THz region was obtained from the velocity autocorrelation function specifically of the alkynyl proton only. The computed VDOS thus represents intermolecular modes that are linked to the phonon modes associated with the C(sp)–H moiety. Moreover, the center gravity of the THz H-bond stretch mode is strongly linearly correlated ($R^2 > 0.88$) with the simulated frequency of the band. This experiment-theory correspondence depicted in Fig. 6c provides another compelling piece of evidence for the specific hydrogen bond associated modes.

Concentration effects

THz measurements were conducted at much higher concentrations (by a factor of ~20 resulting in concentration of ~2 M) than the rest of the experiments. This is inevitable due to intrinsically much lower THz absorption cross-sections. We emphasize that the majority of experiments conducted in this work, especially in volatile solvents (e.g., acetone, THF, chloroform etc.), have proven impossible for a conventional realization of time-domain THz spectroscopy experiment. Extensive averaging over many "slow" scans, where each scan is done in a step-scan mode, takes significant amount of time that exceeds the lifetime of the sample integrity. Each of our samples typically contains several tens of microliters of solution as a thin film of liquid (from 50 micron to 360 micron thick depending on the solvent, see **Section S1.4.3**) held between two sapphire windows that may or may not be wrapped in parafilm around the edge to prevent evaporation. Only due to a set of innovations achieved over the recent few years and described in **Section S1.4.3** we were able to collect these spectra in a fast (1-2 minutes per sample) and efficient manner. Due to all these constraints, the sample concentration had to be increased. However, there are three major pieces of evidence that concentration effects do not play significant role in determining the shape of presented interaction spectra:

i) Aggregation of **MP** is problematic only in non-polar and weakly polar solvents, such as hydrocarbons and lowest-polar ethers, where the effects on the IR spectra were observed. It is much less prone to aggregation in polar media, where **MP** is well-solvated.
ii) Even when present, aggregates THz signature is similar to that of neat **MP** and is largely subtracted by $x_{solute}\alpha_{solute}$ term. The fact that both cyclohexane and isooctane, where aggregation at this concentration is inevitable, nevertheless feature near-zero interaction spectra (**Fig. 6a**) supports this argument.
iii) In non-volatile DMSO, where the sample can be well-evacuated to prevent evaporation, and sample integrity in the cell can be guaranteed for as long as several hours, we were able to carry out several additional experiments on lower concentrations to demonstrate that the shape does not vary, and only the amplitude scales with concentration (**Fig. S20**).

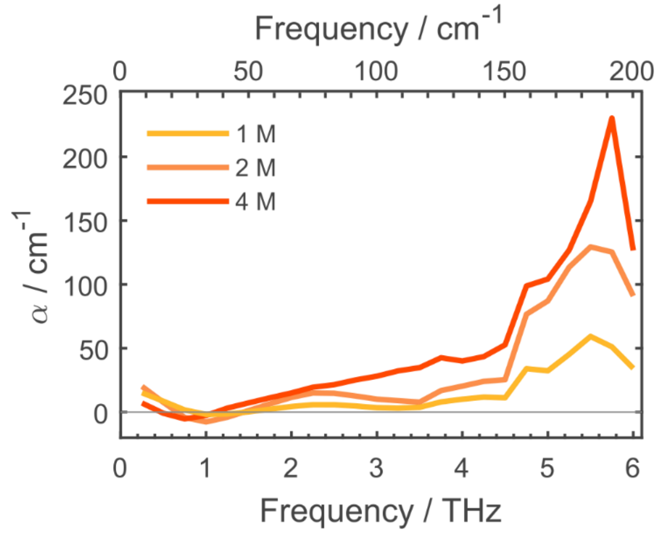

Figure S20. Experimental THz interaction spectra of **MP** in DMSO at three different concentrations. These control experiments were conducted with a conventional step-scan mode using a 5 kHz Ti:Sapphire amplified laser system and a single-channel detection scheme.[S49]

S3 ADDITIONAL SIMULATIONS DATA AND DETAILS

Table S8. Total interaction energy and its physical components (in kcal/mol) obtained from the SAPT calculations (described in **Section S.1.4.5**) for **MP** interacting with solvents from two chemical classes differing in their softness/hardness as discussed in the main text: ethyl acetate (ester) and THF (ether).

Energy	MP+THF	MP+EtAc
Electrostatics	-6.50	-6.16
Exchange	7.18	5.80
Induction	-2.01	-1.81
Dispersion	-3.19	-2.63
**Total**	**-4.52**	**-4.80**

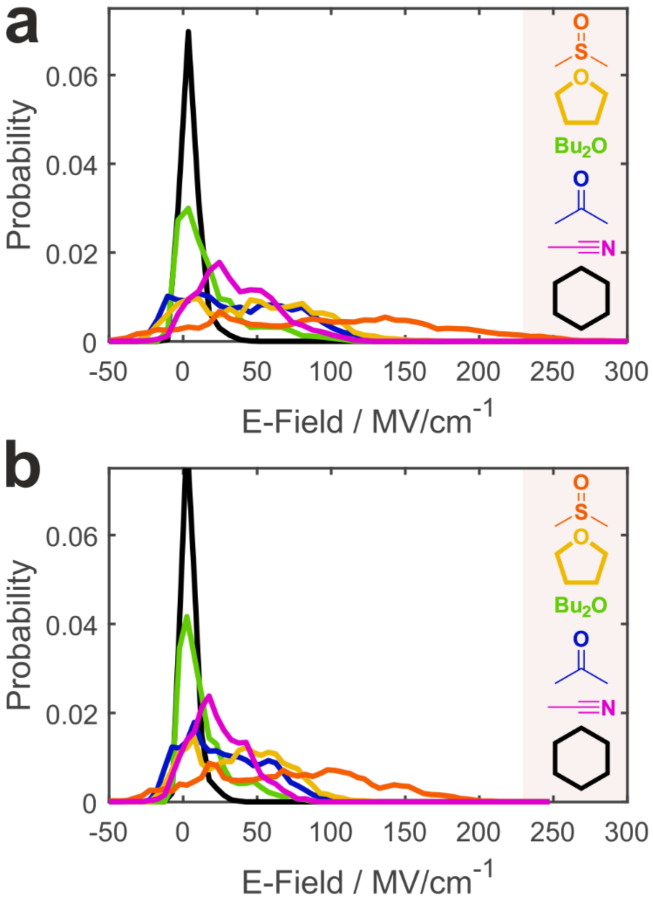

Figure S21. The distribution of electric fields, due to solvent, calculated at the alkynyl H atom and projected onto C(*sp*)–H bond as explained in **S1.4.4** using (**a**) ESP derived and (**b**) Mulliken partial atomic charges.

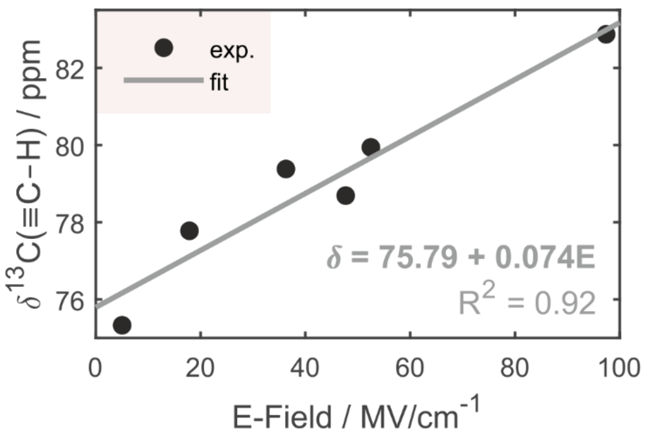

Figure S22. Experimental ^{13}C NMR chemical shift of alkynyl ≡C–H versus electric field, calculated at the alkynyl H atom, and projected onto the C(*sp*)–H bond. Experimental data points are shown as markers, linear fit with its respective coefficient of determination and fit equation is indicated.

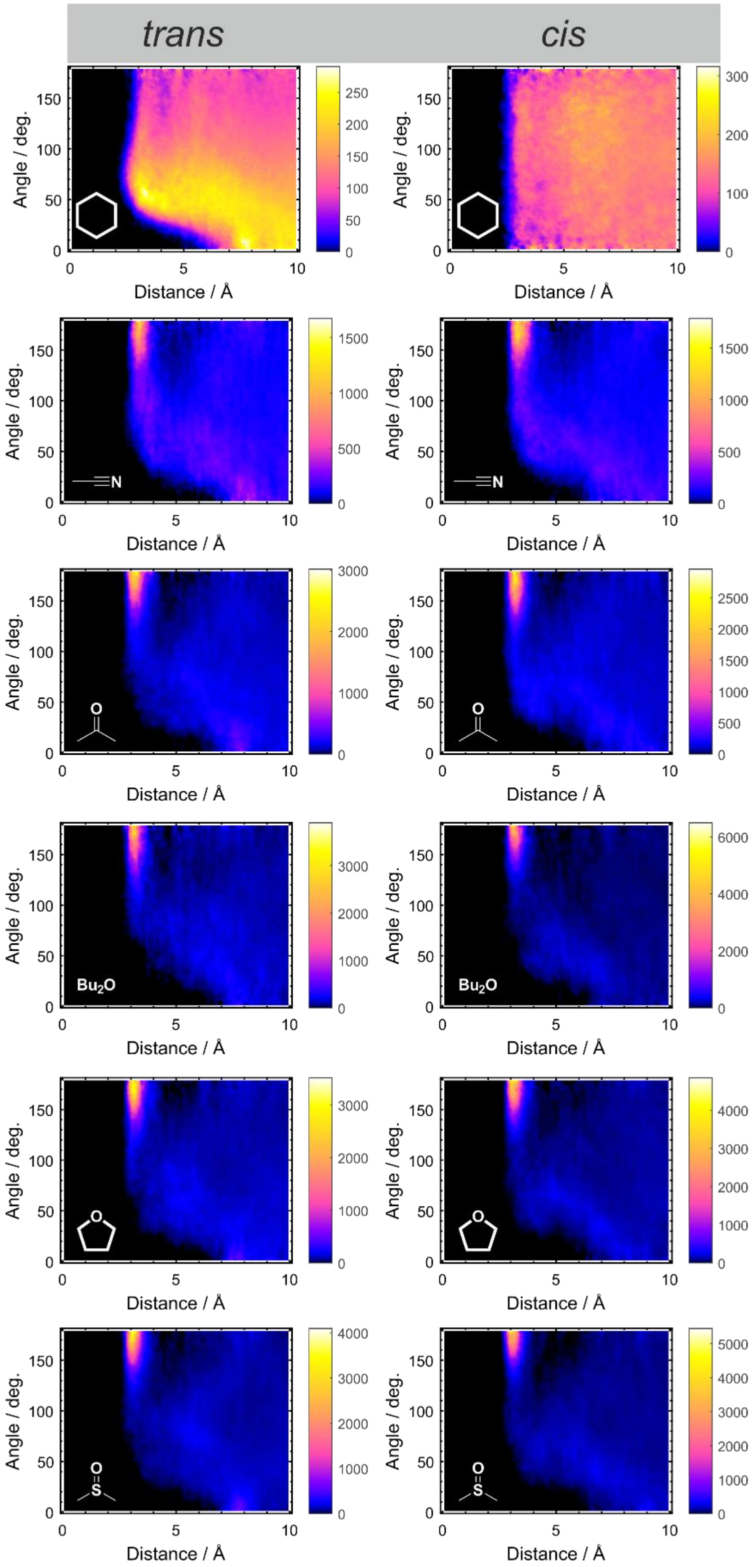

Figure S23. 2D spatial distribution function of C···**A** distance versus C–H···**A** angle from AIMD simulations across all simulated solvents. Left column shows these data for *trans*- conformation of **MP**, while right column shows them for the *cis*- conformation. There is no qualitative difference in behavior or intermolecular interactions regardless of the conformation of **MP**.

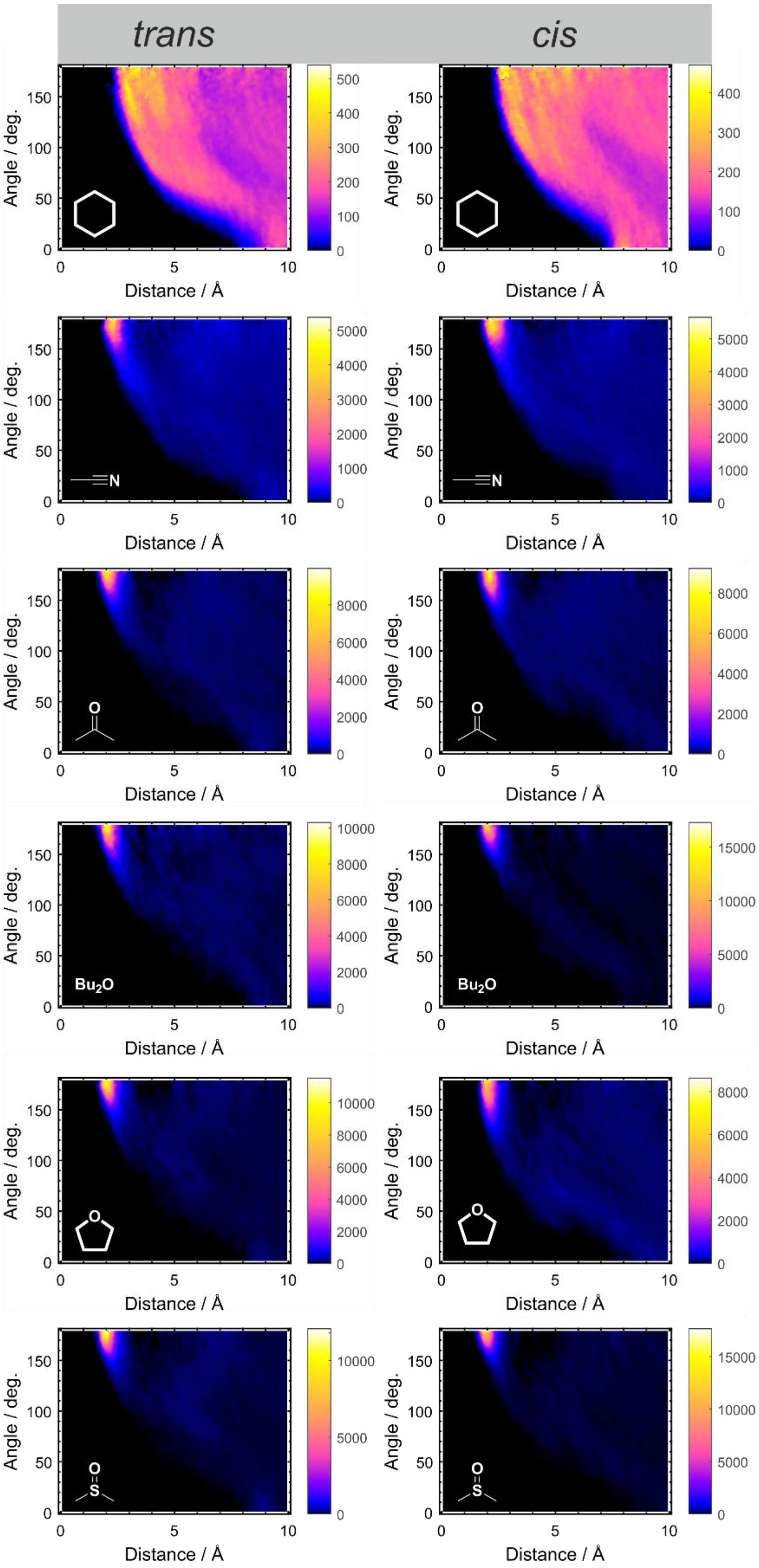

Figure S24. 2D spatial distribution function of H···**A** distance versus C–H···**A** angle from AIMD simulations across all simulated solvents. Left column shows these data for *trans*- conformation of **MP**, while right column shows them for the *cis*- conformation. There is no qualitative difference in behavior or intermolecular interactions regardless of the conformation of **MP**.

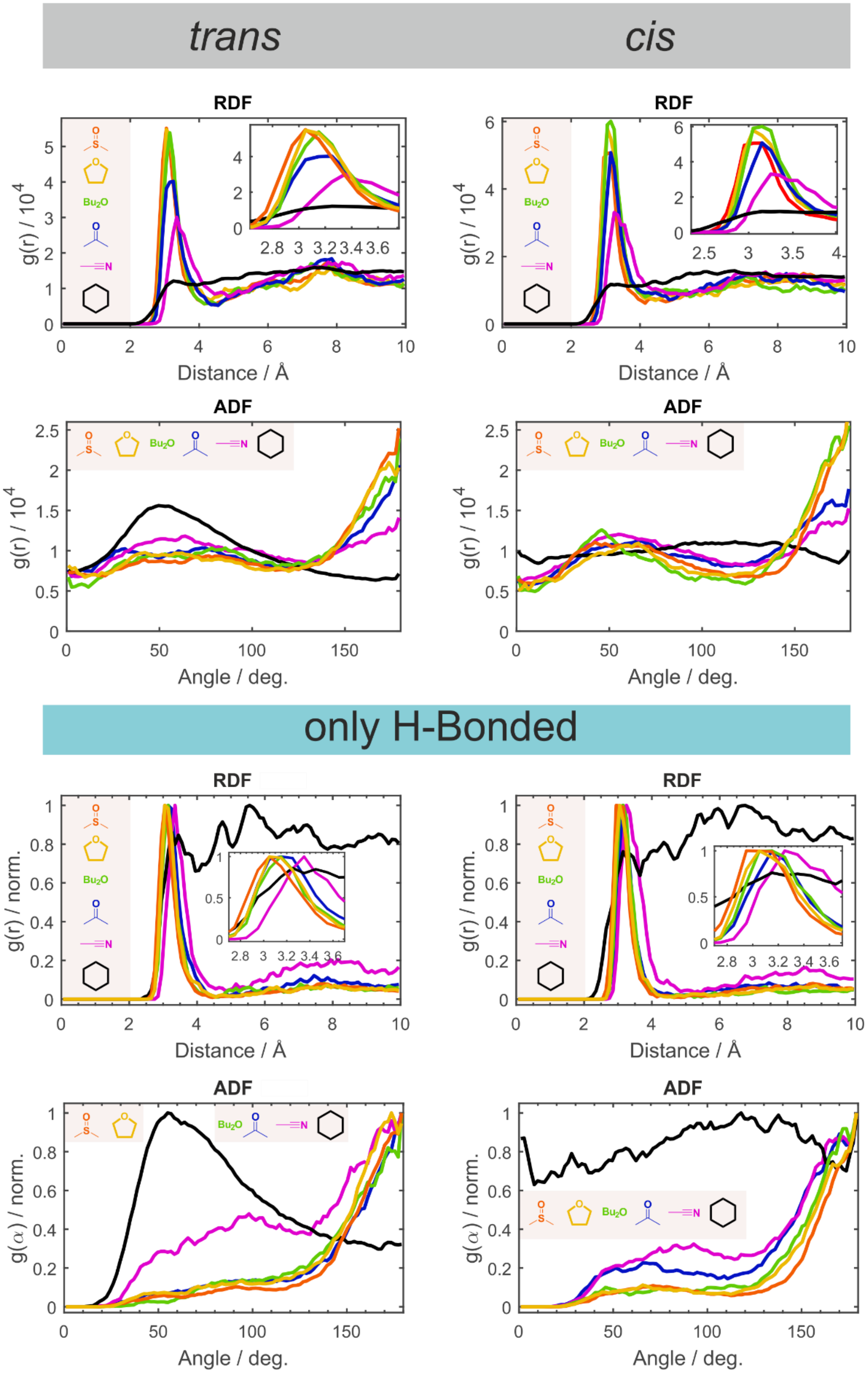

Figure S25. Radial (RDF) and angular (ADF) distribution functions for *cis*- and *trans*-**MP** in all simulated solvents.

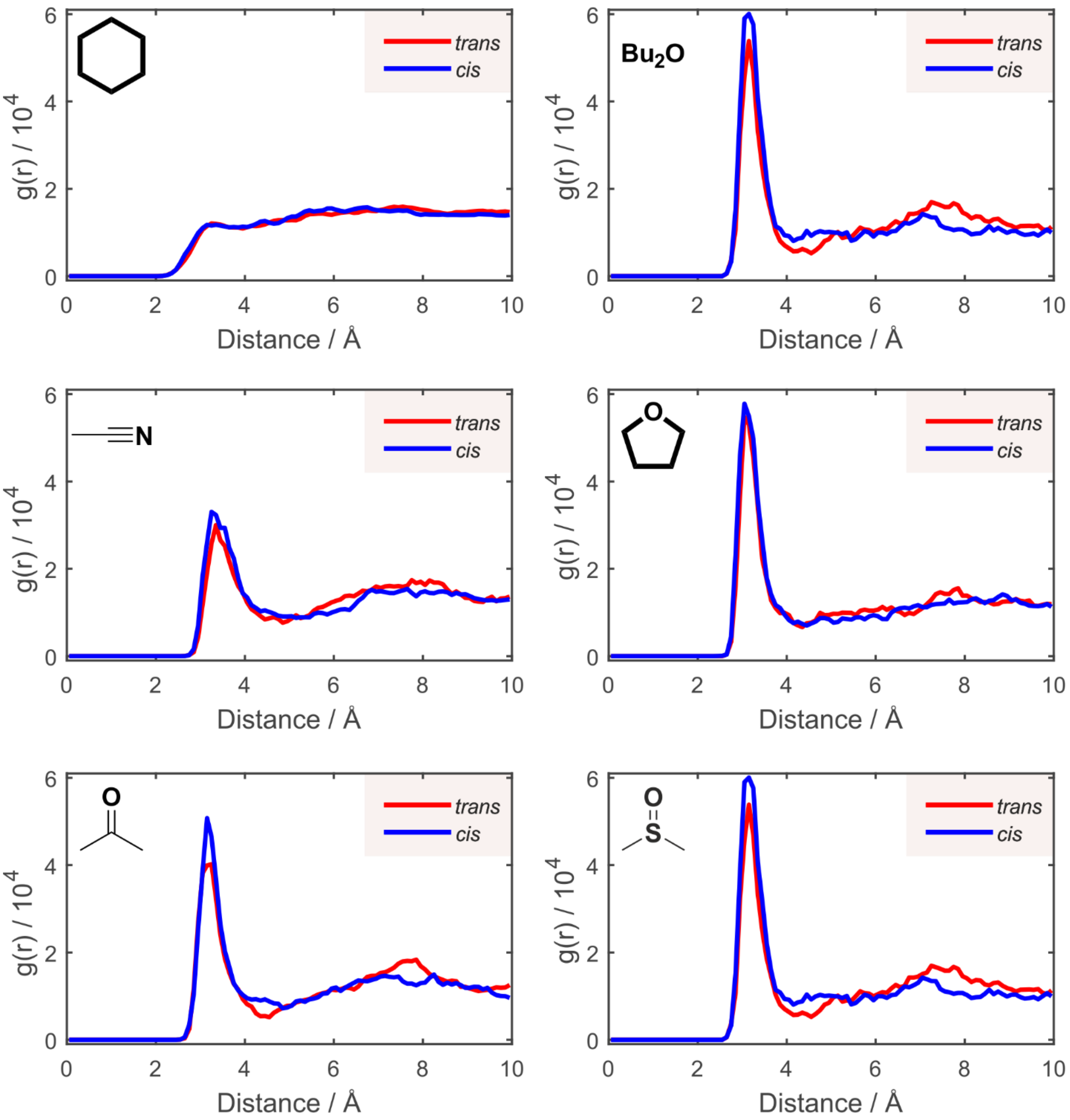

Figure S26. Radial distribution functions for *cis*- and *trans*- conformers of **MP** in all simulated solvents.

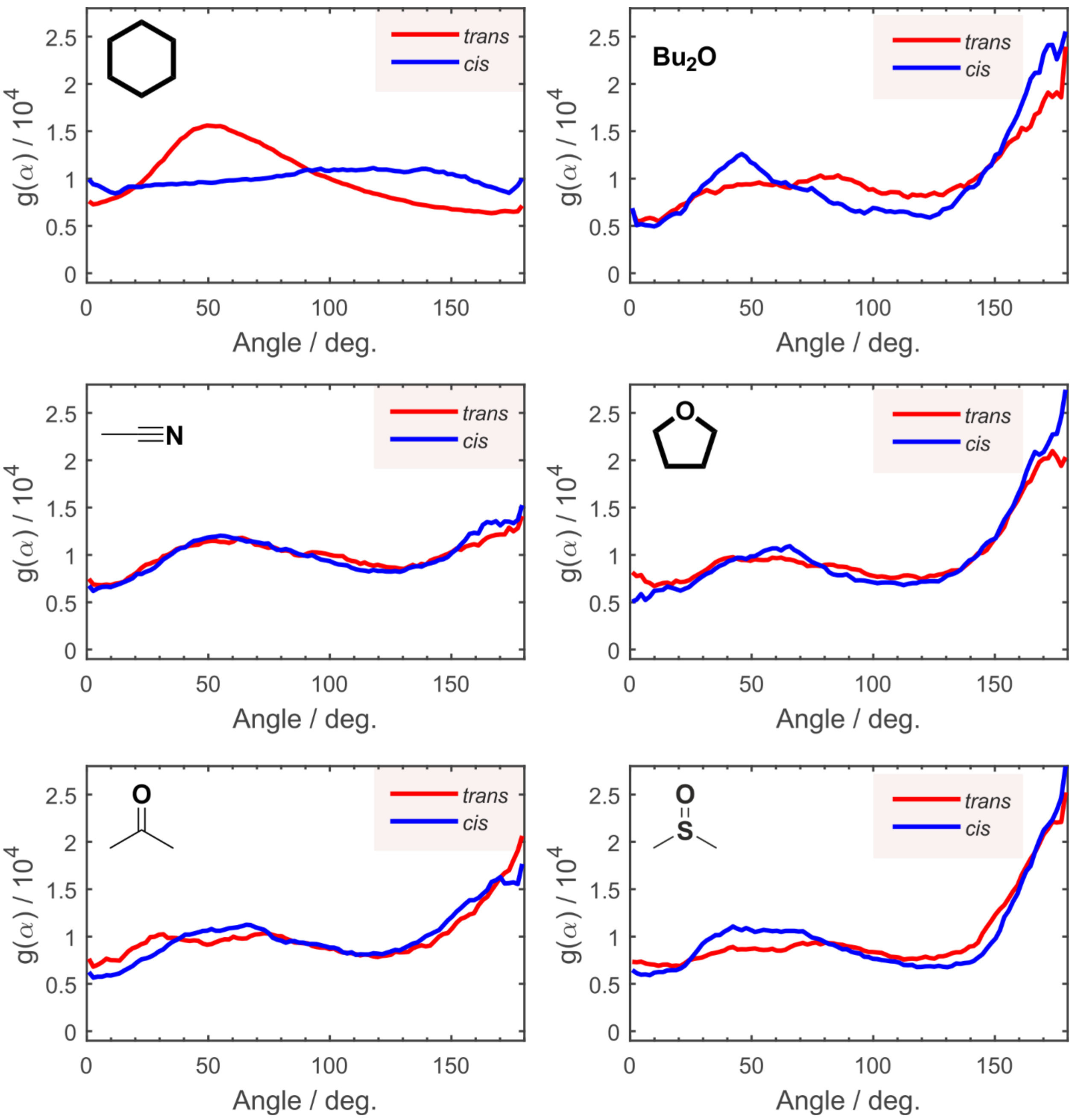

Figure S27. Angular distribution functions for *cis*- and *trans*- conformers of **MP** in all simulated solvents.

S4 SUPPLEMENTARY REFERENCES

(S1) Vidal, S. Safety First: A Recent Case of a Dichloromethane Injection Injury. *ACS Cent Sci* **2020**, *6* (2), 83–86. https://doi.org/10.1021/acscentsci.0c00100.

(S2) *https://www.fishersci.com/store/msds?partNumber=SB01817EA&productDescription=METHYL+PROPIOLATE10GR&vendorId=VN00092202&countryCode=US&language=en*. Methyl Propiolate Material Safety Data Sheet.

(S3) Bredenbeck, J.; Hamm, P. Versatile Small Volume Closed-Cycle Flow Cell System for Transient Spectroscopy at High Repetition Rates. *Review of Scientific Instruments* **2003**, *74* (6), 3188–3189. https://doi.org/10.1063/1.1574605.

(S4) Shukla, S.; Shalit, A.; Hamm, P. 2D Raman-THz Spectroscopy of Imidazolium-Based Ionic Liquids. *J Chem Phys* **2025**, *162* (3), 034502. https://doi.org/10.1063/5.0246152.

(S5) Duchi, M.; Shukla, S.; Shalit, A.; Hamm, P. 2D-Raman-THz Spectroscopy with Single-Shot THz Detection. *J Chem Phys* **2021**, *155* (17), 174201. https://doi.org/10.1063/5.0065804.

(S6) Farrell, K. M.; Ostrander, J. S.; Jones, A. C.; Yakami, B. R.; Dicke, S. S.; Middleton, C. T.; Hamm, P.; Zanni, M. T. Shot-to-Shot 2D IR Spectroscopy at 100 kHz Using a Yb Laser and Custom-Designed Electronics. *Opt Express* **2020**, *28* (22), 33584. https://doi.org/10.1364/OE.409360.

(S7) Mousavi, S. J.; Biggs, M. F.; Johnson, J. A.; Hamm, P.; Shalit, A. $\chi^{(2)}$-Induced Artifact Overwhelming the Third-Order Signal in 2D Raman–THz Spectroscopy of Non-Centrosymmetric Materials. *J Chem Phys* **2024**, *161* (13), 134502. https://doi.org/10.1063/5.0228378.

(S8) Johnson, J. A.; Brunner, F. D. J.; Grübel, S.; Ferrer, A.; Johnson, S. L.; Feurer, T. Distortion-Free Enhancement of Terahertz Signals Measured by Electro-Optic Sampling II Experiment. *Journal of the Optical Society of America B* **2014**, *31* (5), 1035. https://doi.org/10.1364/JOSAB.31.001035.

(S9) Feng, Y.; Vinogradov, I.; Ge, N.-H. Optimized Noise Reduction Scheme for Heterodyne Spectroscopy Using Array Detectors. *Opt Express* **2019**, *27* (15), 20323. https://doi.org/10.1364/OE.27.020323.

(S10) Withayachumnankul, W.; Naftaly, M. Fundamentals of Measurement in Terahertz Time-Domain Spectroscopy. *J Infrared Millim Terahertz Waves* **2014**, *35* (8), 610–637. https://doi.org/10.1007/s10762-013-0042-z.

(S11) Chai, J.-D.; Head-Gordon, M. Long-Range Corrected Hybrid Density Functionals with Damped Atom–Atom Dispersion Corrections. *Physical Chemistry Chemical Physics* **2008**, *10* (44), 6615. https://doi.org/10.1039/b810189b.

(S12) Weigend, F.; Ahlrichs, R. Balanced Basis Sets of Split Valence, Triple Zeta Valence and Quadruple Zeta Valence Quality for H to Rn: Design and Assessment of Accuracy. *Physical Chemistry Chemical Physics* **2005**, *7* (18), 3297. https://doi.org/10.1039/b508541a.

(S13) Frisch, M. J.; Trucks, G. W.; Schlegel, H. B.; Scuseria, G. E.; Robb, M. A.; Cheeseman, J. R.; Scalmani, G.; Barone, V.; Petersson, G. A.; Nakatsuji, H.; Li, X.; Caricato, M.; Marenich, A. V.; Bloino, J.; Janesko, B. G.; Gomperts, R.; Mennucci, B.; Hratchian, H. P.; Ortiz, J. V.; Izmaylov, A. F.; Sonnenberg, J. L.; Williams-Young, D.; Ding, F.; Lipparini, F.; Egidi, F.; Goings, J.; Peng, B.;

Petrone, A.; Henderson, T.; Ranasinghe, D.; Zakrzewski, V. G.; Gao, J.; Rega, N.; Zheng, G.; Liang, W.; Hada, M.; Ehara, M.; Toyota, K.; Fukuda, R.; Hasegawa, J.; Ishida, M.; Nakajima, T.; Honda, Y.; Kitao, O.; Nakai, H.; Vreven, T.; Throssell, K.; Montgomery, J. A., Jr.; Peralta, J. E.; Ogliaro, F.; Bearpark, M. J.; Heyd, J. J.; Brothers, E. N.; Kudin, K. N.; Staroverov, V. N.; Keith, T. A.; Kobayashi, R.; Normand, J.; Raghavachari, K.; Rendell, A. P.; Burant, J. C.; Iyengar, S. S.; Tomasi, J.; Cossi, M.; Millam, J. M.; Klene, M.; Adamo, C.; Cammi, R.; Ochterski, J. W.; Martin, R. L.; Morokuma, K.; Farkas, O.; Foresman, J. B.; Fox, D. J. Gaussian 16 (Revision A.03). Gaussian, Inc.: Wallingford CT 2016.

(S14) Hehre, W. J.; Ditchfield, R.; Pople, J. A. Self—Consistent Molecular Orbital Methods. XII. Further Extensions of Gaussian—Type Basis Sets for Use in Molecular Orbital Studies of Organic Molecules. *J Chem Phys* **1972**, *56* (5), 2257–2261. https://doi.org/10.1063/1.1677527.

(S15) Hariharan, P. C.; Pople, J. A. The Influence of Polarization Functions on Molecular Orbital Hydrogenation Energies. *Theor Chim Acta* **1973**, *28* (3), 213–222. https://doi.org/10.1007/BF00533485.

(S16) Papajak, E.; Truhlar, D. G. Efficient Diffuse Basis Sets for Density Functional Theory. *J Chem Theory Comput* **2010**, *6* (3), 597–601. https://doi.org/10.1021/ct900566x.

(S17) Martínez, L.; Andrade, R.; Birgin, E. G.; Martínez, J. M. PACKMOL: A Package for Building Initial Configurations for Molecular Dynamics Simulations. *J Comput Chem* **2009**, *30* (13), 2157–2164. https://doi.org/10.1002/jcc.21224.

(S18) Pronk, S.; Páll, S.; Schulz, R.; Larsson, P.; Bjelkmar, P.; Apostolov, R.; Shirts, M. R.; Smith, J. C.; Kasson, P. M.; van der Spoel, D.; Hess, B.; Lindahl, E. GROMACS 4.5: A High-Throughput and Highly Parallel Open Source Molecular Simulation Toolkit. *Bioinformatics* **2013**, *29* (7), 845–854. https://doi.org/10.1093/bioinformatics/btt055.

(S19) Payne, M. C.; Teter, M. P.; Allan, D. C.; Arias, T. A.; Joannopoulos, J. D. Iterative Minimization Techniques for Ab Initio Total-Energy Calculations: Molecular Dynamics and Conjugate Gradients. *Rev Mod Phys* **1992**, *64* (4), 1045–1097. https://doi.org/10.1103/RevModPhys.64.1045.

(S20) Jorgensen, W. L.; Maxwell, D. S.; Tirado-Rives, J. Development and Testing of the OPLS All-Atom Force Field on Conformational Energetics and Properties of Organic Liquids. *J Am Chem Soc* **1996**, *118* (45), 11225–11236. https://doi.org/10.1021/ja9621760.

(S21) Essmann, U.; Perera, L.; Berkowitz, M. L.; Darden, T.; Lee, H.; Pedersen, L. G. A Smooth Particle Mesh Ewald Method. *J Chem Phys* **1995**, *103* (19), 8577–8593. https://doi.org/10.1063/1.470117.

(S22) Darden, T.; York, D.; Pedersen, L. Particle Mesh Ewald: An N·log(N) Method for Ewald Sums in Large Systems. *J Chem Phys* **1993**, *98* (12), 10089–10092. https://doi.org/10.1063/1.464397.

(S23) York, D. M.; Darden, T. A.; Pedersen, L. G. The Effect of Long-Range Electrostatic Interactions in Simulations of Macromolecular Crystals: A Comparison of the Ewald and Truncated List Methods. *J Chem Phys* **1993**, *99* (10), 8345–8348. https://doi.org/10.1063/1.465608.

(S24) Verlet, L. Computer "Experiments" on Classical Fluids. I. Thermodynamical Properties of Lennard-Jones Molecules. *Physical Review* **1967**, *159* (1), 98–103. https://doi.org/10.1103/PhysRev.159.98.

(S25) VandeVondele, J.; Krack, M.; Mohamed, F.; Parrinello, M.; Chassaing, T.; Hutter, J. Quickstep: Fast and Accurate Density Functional Calculations Using a Mixed Gaussian and Plane Waves

Approach. *Comput Phys Commun* **2005**, *167* (2), 103–128. https://doi.org/10.1016/j.cpc.2004.12.014.

(S26) Kühne, T. D.; Iannuzzi, M.; Del Ben, M.; Rybkin, V. V.; Seewald, P.; Stein, F.; Laino, T.; Khaliullin, R. Z.; Schütt, O.; Schiffmann, F.; Golze, D.; Wilhelm, J.; Chulkov, S.; Bani-Hashemian, M. H.; Weber, V.; Borštnik, U.; Taillefumier, M.; Jakobovits, A. S.; Lazzaro, A.; Pabst, H.; Müller, T.; Schade, R.; Guidon, M.; Andermatt, S.; Holmberg, N.; Schenter, G. K.; Hehn, A.; Bussy, A.; Belleflamme, F.; Tabacchi, G.; Glöß, A.; Lass, M.; Bethune, I.; Mundy, C. J.; Plessl, C.; Watkins, M.; VandeVondele, J.; Krack, M.; Hutter, J. CP2K: An Electronic Structure and Molecular Dynamics Software Package - Quickstep: Efficient and Accurate Electronic Structure Calculations. *J Chem Phys* **2020**, *152* (19), 194103. https://doi.org/10.1063/5.0007045.

(S27) Becke, A. D. Density-Functional Thermochemistry. III. The Role of Exact Exchange. *J Chem Phys* **1993**, *98* (7), 5648–5652. https://doi.org/10.1063/1.464913.

(S28) Lee, C.; Yang, W.; Parr, R. G. Development of the Colle-Salvetti Correlation-Energy Formula into a Functional of the Electron Density. *Phys Rev B* **1988**, *37* (2), 785–789. https://doi.org/10.1103/PhysRevB.37.785.

(S29) Grimme, S.; Antony, J.; Ehrlich, S.; Krieg, H. A Consistent and Accurate Ab Initio Parametrization of Density Functional Dispersion Correction (DFT-D) for the 94 Elements H-Pu. *J Chem Phys* **2010**, *132* (15), 154104. https://doi.org/10.1063/1.3382344.

(S30) Goedecker, S.; Teter, M.; Hutter, J. Separable Dual-Space Gaussian Pseudopotentials. *Phys Rev B* **1996**, *54* (3), 1703–1710. https://doi.org/10.1103/PhysRevB.54.1703.

(S31) Hartwigsen, C.; Goedecker, S.; Hutter, J. Relativistic Separable Dual-Space Gaussian Pseudopotentials from H to Rn. *Phys Rev B* **1998**, *58* (7), 3641–3662. https://doi.org/10.1103/PhysRevB.58.3641.

(S32) Eichinger, M.; Tavan, P.; Hutter, J.; Parrinello, M. A Hybrid Method for Solutes in Complex Solvents: Density Functional Theory Combined with Empirical Force Fields. *J Chem Phys* **1999**, *110* (21), 10452–10467. https://doi.org/10.1063/1.479049.

(S33) Barbieri, P. L.; Fantin, P. A.; Jorge, F. E. Gaussian Basis Sets of Triple and Quadruple Zeta Valence Quality for Correlated Wave Functions. *Mol Phys* **2006**, *104* (18), 2945–2954. https://doi.org/10.1080/00268970600899018.

(S34) Peintinger, M. F.; Oliveira, D. V.; Bredow, T. Consistent Gaussian Basis Sets of Triple-zeta Valence with Polarization Quality for Solid-state Calculations. *J Comput Chem* **2013**, *34* (6), 451–459. https://doi.org/10.1002/jcc.23153.

(S35) Evans, D. J.; Holian, B. L. The Nose–Hoover Thermostat. *J Chem Phys* **1985**, *83* (8), 4069–4074. https://doi.org/10.1063/1.449071.

(S36) Shelly, R. A.; Toprak, K.; Bayazitoglu, Y. Nose–Hoover Thermostat Length Effect on Thermal Conductivity of Single Wall Carbon Nanotubes. *Int J Heat Mass Transf* **2010**, *53* (25–26), 5884–5887. https://doi.org/10.1016/j.ijheatmasstransfer.2010.06.054.

(S37) Singh, U. C.; Kollman, P. A. An Approach to Computing Electrostatic Charges for Molecules. *J Comput Chem* **1984**, *5* (2), 129–145. https://doi.org/10.1002/jcc.540050204.

(S38) Besler, B. H.; Merz, K. M.; Kollman, P. A. Atomic Charges Derived from Semiempirical Methods. *J Comput Chem* **1990**, *11* (4), 431–439. https://doi.org/10.1002/jcc.540110404.

(S39) Mulliken, R. S. Electronic Population Analysis on LCAO–MO Molecular Wave Functions. I. *J Chem Phys* **1955**, *23* (10), 1833–1840. https://doi.org/10.1063/1.1740588.

(S40) Mennucci, B. Polarizable Continuum Model. *WIREs Computational Molecular Science* **2012**, *2* (3), 386–404. https://doi.org/10.1002/wcms.1086.

(S41) Jeziorski, B.; Moszynski, R.; Szalewicz, K. Perturbation Theory Approach to Intermolecular Potential Energy Surfaces of van Der Waals Complexes. *Chem Rev* **1994**, *94* (7), 1887–1930. https://doi.org/10.1021/cr00031a008.

(S42) Møller, Chr.; Plesset, M. S. Note on an Approximation Treatment for Many-Electron Systems. *Physical Review* **1934**, *46* (7), 618–622. https://doi.org/10.1103/PhysRev.46.618.

(S43) Feyereisen, M.; Fitzgerald, G.; Komornicki, A. Use of Approximate Integrals in Ab Initio Theory. An Application in MP2 Energy Calculations. *Chem Phys Lett* **1993**, *208* (5–6), 359–363. https://doi.org/10.1016/0009-2614(93)87156-W.

(S44) Kendall, R. A.; Dunning, T. H.; Harrison, R. J. Electron Affinities of the First-Row Atoms Revisited. Systematic Basis Sets and Wave Functions. *J Chem Phys* **1992**, *96* (9), 6796–6806. https://doi.org/10.1063/1.462569.

(S45) Parker, T. M.; Burns, L. A.; Parrish, R. M.; Ryno, A. G.; Sherrill, C. D. Levels of Symmetry Adapted Perturbation Theory (SAPT). I. Efficiency and Performance for Interaction Energies. *J Chem Phys* **2014**, *140* (9), 094106. https://doi.org/10.1063/1.4867135.

(S46) Smith, D. G. A.; Burns, L. A.; Simmonett, A. C.; Parrish, R. M.; Schieber, M. C.; Galvelis, R.; Kraus, P.; Kruse, H.; Di Remigio, R.; Alenaizan, A.; James, A. M.; Lehtola, S.; Misiewicz, J. P.; Scheurer, M.; Shaw, R. A.; Schriber, J. B.; Xie, Y.; Glick, Z. L.; Sirianni, D. A.; O'Brien, J. S.; Waldrop, J. M.; Kumar, A.; Hohenstein, E. G.; Pritchard, B. P.; Brooks, B. R.; Schaefer, H. F.; Sokolov, A. Y.; Patkowski, K.; DePrince, A. E.; Bozkaya, U.; King, R. A.; Evangelista, F. A.; Turney, J. M.; Crawford, T. D.; Sherrill, C. D. PSI4 1.4: Open-Source Software for High-Throughput Quantum Chemistry. *J Chem Phys* **2020**, *152* (18), 184108. https://doi.org/10.1063/5.0006002.

(S47) Nyquist, R. A.; Potts, W. J. Infrared Absorptions Characteristic of the Terminal Acetylenic Group (-C≡C-H). *Spectrochimica Acta* **1960**, *16* (4), 419–427. https://doi.org/10.1016/0371-1951(60)80036-7.

(S48) Zheng, C.; Mao, Y.; Markland, T. E.; Boxer, S. G. Beyond the Vibrational Stark Effect: Unraveling the Large Redshifts of Alkyne C–H Bond in Solvation Environments. *J Am Chem Soc* **2025**, *147* (7), 6227–6235. https://doi.org/10.1021/jacs.4c18102.

(S49) Mousavi S. J.; Berger, A.; Hamm, P.; Shalit, A. Low-frequency Anharmonic Couplings in Bromoform Revealed from 2D Raman-THz Spectroscopy: from the Liquid to the Crystalline Phase. *J. Chem. Phys.* **2022**, 156, 174501. https://doi.org/10.1063/5.0090520.
